# Blocking interplay between TERT and c-Myc: a new therapeutic strategy for *BRAF^V600E^/pTERT* double mutated tumors

**DOI:** 10.7150/ijbs.111224

**Published:** 2025-07-28

**Authors:** Jing Wei, Jiazhe Liu, Yan Zhang, Guobin Wang, Xiao Cui, Wei Yu, Chunlei Nie, Peng Hou

**Affiliations:** 1 Department of Endocrinology and Metabolism, The First Affiliated Hospital of Xi'an Jiaotong University, Xi'an 710061, P.R. China.; 2 International Joint Research Center for Tumor Precision Medicine of Shaanxi Province, The First Affiliated Hospital of Xi'an Jiaotong University, Xi'an 710061, P.R. China.; 3 Department of Basic Medicine, Shaanxi University of Chinese Medicine, Xianyang 712046, P.R. China; 4 BioBank, The First Affiliated Hospital of Xi'an Jiaotong University, Xi'an 710061, P.R. China; 5 Center for Biobank and Advanced Medical Research of Shaanxi Province, The First Affiliated Hospital of Xi'an Jiaotong University, Xi'an 710061, P.R. China; 6 Department of Head and Neck Surgery, Harbin Medical University Cancer Hospital, Harbin 163711, P.R. China

**Keywords:** *BRAF^V600E^* mutation, *pTERT* mutations, c-Myc stability, Peptide-gold nanoparticles

## Abstract

Tumors with coexisting mutations of *BRAF^V600E^* and *TERT* promoter (*pTERT*) are more aggressive and associated with poor patient survival. However, the effective treatments for these tumors are limited, and the mutual regulation mechanism between these two molecules remains largely unclear. Here, we demonstrated that BRAF and TERT could mutually regulate each other, and c-Myc played a vital role in this process. Mechanistically, c-Myc could promote *BRAF* transcription*,* and TERT interacted with and stabilized c-Myc. Meanwhile, we verified that c-Myc transcriptionally repressed *PP2Ac*, which, as the core catalytic subunit of PP2A, leads to dephosphorylation of c-Myc at Ser62, decreasing its stability. These molecular events promoted the progression of *BRAF^V600E^/pTERT* double mutated tumors by forming positive regulatory networks. To develop therapeutic strategy for this kind of tumors, we designed two peptides p-CPS62 and CPS62 to break the interaction between TERT and c-Myc, and constructed the corresponding aurous nanoparticles (AuNP-p-CPS62 and AuNP-CPS62). The results showed that AuNP-p-CPS62 and AuNP-CPS62, especially the former, effectively suppressed the growth of *BRAF^V600E^/pTERT* double mutated cancer cells both *in vitro* and *vivo*, with good biosafety. These findings suggest that blocking the interaction between TERT and c-Myc may be a promising therapeutic option for *BRAF^V600E^/pTERT* double mutated tumors.

## Introduction

Telomerase reverse transcriptase (TERT) is the essential catalytic subunit of human telomerase 1, responsible for extending telomere length to preserve chromosomal integrity and maintain genome stability 2. The frequently mutant *TERT* promoter (*pTERT*) forms de novo ETS family transcription factor binding sites and increases transcriptional activity in cancer cells by selectively recruiting ETS transcription factors, such as GABPA 3, ultimately activating telomerase and leading to cell immortalization 4. Patients with *pTERT* mutations are associated with poor overall survival in various cancers, including glioma 5, thyroid cancer 6-8, melanoma 9, breast cancer 10 and bladder cancer 11. In addition, TERT also acts as a transcriptional modulator to regulate PI3K/AKT, Wnt/β-catenin and NF-kB signaling pathways, thereby promoting tumor progression 12. Inhibiting telomerase significantly enhances the therapeutic effect of radiation on glioma, lung cancer and other tumors 13.

*BRAF^V600E^* mutation is another frequent genetic event that drives tumorigenesis and tumor progression by activating MAPK/ERK signaling pathway 14. The patients with *BRAF^V600E^* mutation are insensitive to BRAF kinase inhibitors which is caused by the inevitable cross drug resistance and severe adverse effects 15. Additionally, the high-frequency coexisting mutations of *BRAF^V600E^* and *pTERT* in anaplastic thyroid cancer (ATC) and melanoma are strongly associated with high tumor node metastasis (TNM) staging 16, poor patient survival 17, increased risk of recurrence and limited therapies 18-20, suggesting the synergistic effect between BRAF and TERT on tumorigenesis. Evidently, *BRAF^V600E^* mutation can enhance the transcription of *TERT* by phosphorylating Sp1 and promote the formation of GABP complex on the mutant *pTERT*, ultimately up-regulating TERT expression 21. However, it is obscure whether TERT is involved in the regulation of *BRAF* gene. Therefore, exploring the regulatory relationship between *BRAF^V600E^* and *pTERT* mutations and developing effective therapeutic strategies are major challenges in *BRAF^V600E^/pTERT* double mutated tumors.

The c-Myc is a “master regulator” that is the most commonly activated oncoproteins in human cancers 22, 23. The key features of c-Myc oncogenicity include chromosomal amplification, translocations or mutations 24. As a transcription factor hub, c-Myc regulates approximately 15% of human genes and play an essential role in multiple cellular processes 25. The activation of c-Myc has been widely reported in various cancers, including glioma, thyroid cancer, breast cancer, lymphoma and others 26, 27. c-Myc contains several conserved regions (MBI-IV), which locates within the core transactivation domain (TAD) 24, while c-Myc stability can be regulated by ubiquitylation and proteolysis in MBI region. Activated ERK phosphorylates c-Myc at serine 62 (Ser62) to enhance its protein stability, whereas GSK-3β phosphorylates c-Myc at threonine 58 (Thr58) and trigger Ser62 dephosphorylation via PP2A. This process is followed by Fbw7 E3 ligase-mediated ubiquitylation, ultimately leading to proteasomal degradation of c-Myc 28. However, c-Myc is undruggable due to the lacking of accessible drug recognition sites, leading to current therapies are nonspecific and ineffective 29. c-Myc has also been demonstrated to be involved in drug resistance to MAPK/ERK pathway inhibitors 30. Meanwhile, c-Myc can directly regulate the transcription of *TERT* regardless of the presence or absence of *pTERT* mutations. In addition, there are evidence showing that *BRAF^V600E^* mutation promotes transcription of c-Myc via MAPK/ERK pathway 31, and TERT enhances the protein stability of c-Myc by directly binding to its MBI region 32. Thus, there is a complex regulatory network among *BRAF^V600E^*, TERT and c-Myc.

Intracellular protein-protein interactions (PPIs) are inadequately exploited class of therapeutic targets, which play critical roles in the development and progression of human diseases including malignancies 33. The chemical and structural diversity of peptides ensure their perfect binding to the interacting surfaces of PPIs, thus effectively blocking PPIs 34. Furthermore, aurous nanoparticles (AuNPs) have been successfully used as nanocarrier to deliver targeted anti-cancer drugs including peptides 35, especially for the treatment of solid tumors 36. Therefore, new treatments based on AuNPs-loaded peptides will be highly expected in the precise therapy of tumors. In the present study, we revealed that *BRAF^V600E^* and *pTERT* mutations had a mutual regulatory relationship, and c-Myc played a central role in this process. Moreover, we demonstrated that TERT interacted with and stabilized c-Myc, and identified that *BRAF, TERT* and *PP2Ac* were downstream targets of c-Myc, forming a complex regulatory network among BRAF, TERT and c-Myc. Thus, we developed peptide-gold nanoparticles to induce c-Myc degradation by blocking the interaction between TERT and c-Myc, effectively killing *BRAF^V600E^/pTERT* double mutated cancer cells.

## Materials and Methods

### Cell culture and drug treatment

Human thyroid cancer cell lines 8305C and BCPAP were provided from Dr. Haixia Guan (The First Affiliated Hospital of China Medical University, Shenyang, P.R. China). Human melanoma cell lines A375, M14 and colon cancer cell line RKO were obtained from the American Type Culture Collection (ATCC) (Manassas, VA, USA). Human glioma cell line SF295 was purchased from the Cell Bank of Animal Laboratory Center of Zhongshan University (Guangzhou, China). These cell lines were routinely cultured at 37℃ in RPMI 1640 medium or DMEM medium with 10% fetal bovine serum (FBS). In some experiments, cells were treated with 4 μM BRAF kinase inhibitor PLX4032 (Selleck Chemicals, TX, USA), 25 μM MG132 purchased from Selleck Chemicals (Houston, TX, USA) or 200 µg/ml cycloheximide purchased from MP Biomedicals (Santa Ana, CA, USA). They were dissolved in dimethylsulfoxide (DMSO), aliquoted and stored at -80°C until use. The same volume of DMSO was used as the vehicle control.

### RNA extraction and quantitative RT-PCR (qRT-PCR)

The protocols of RNA extraction, cDNA synthesis and qRT-PCR were described previously 37. The primer sequences were presented in Table S1.

### Western blotting analysis

Western blotting analysis was performed to measure the expression of the indicated proteins as previously described 38. The specific antibodies were shown in Table S2.

### Transfection of short interfering RNAs (siRNAs) and overexpression plasmid

Control siRNA and target-specific siRNAs were purchased from Ribobio (Guangzhou, China), and Table S3 presented their sequences. The open reading frame of c-Myc was cloned into pcDNA3.1(-)A obtained from Yingrun biotechnology, Co., Ltd (Changsha, China). The primers used for plasmid construction were presented in Table S4. siRNAs and overexpression plasmids was transfected as described previously 37.

### Dual-luciferase reporter system

The protocol was similarly described previously 37, and the primers used for plasmid construction were presented in Table S5. All the assays were repeated three times.

### Chromatin immunoprecipitation (ChIP)

ChIP assays were performed using antibody against c-Myc (sc-764, Santa Cruz) according to a previous protocol 37. The data was normalized by respective 5% input. The primers were shown in Table S6.

### Co-immunoprecipitation (Co-IP)

Co-IP was used to evaluate the interaction between TERT and c-Myc based on a previous protocol 38.

### Fabrication of AuNP-Ctr, AuNP-p-CPS62 and AuNP-CPS62

p-CPS62 and CPS62 peptides were synthesized by Gil Biochemical technology Co., Ltd (Shanghai, China), and their sequences were presented in Table S7. AuNPs were prepared by HEPES REDOX method. An aqueous solution of tetra chloroauric acid (HAuCl4·xH2O, 1 mL, 10 mM) was mixed with 9 mL HEPES buffer (pH 7.4, 50 mM). After 10 min of magnetic stirring, the solution color changed from golden yellow to wine red, which was the Au core. To determine the *in vivo* biodistribution of AuNP-p-CPS62 and AuNP-CPS62, 2 mg of Cy5.5 fluorescein-labeled p-CPS62 or CPS62 peptides were added to the Au core solution under stirring to form Au-peptide particles by gold-thiol bonding. Next, the solution was successively mixed with 500 µL polyethylene glycol (PEG) buffer to improve peptide carrying efficiency. Then, 50 µL polyallylamine hydrochloride (PAH buffer) was added to encapsulate Au-peptide particles for further synthesizing Au-peptide-PAH particles, giving nanoparticles better hydrophilicity and more functional groups to bind to targeted molecules. Finally, the solution color changed to bluish violet, indicating that the peptides were successfully conjugated to the Au-core to form AuNP-p-CPS62 and AuNP-CPS62. After 30 min of stirring, the excess reactants were removed by dialysis (cutoff, 10 kDa) and washed three times with PBS buffer. AuNP-p-CPS62 and AuNP-CPS62 were then freeze-dried for subsequent experiments. They could enter the tumor microenvironment and target tumor cells via the EPR effect, triggering the release of peptides through the REDOX cleavage of gold-thiol bonding 39.

### Physicochemical properties of AuNPs

The UV-vis absorption spectroscopy was measured by Shimadzu 3000 spectrophotometer. RP-HPLC analyses were performed on a Waters XBridge C18 column (4.6 × 150 mm, 3.5 μm) at 40℃ in a flow rate of 1 mL/min (5%-65% linear gradient acetonitrilein and 0.1% TFA in water), 30 min. Malvern Zetasizer Nano ZS system was used to obtain the hydrodynamic size distribution of nanoparticle solutions (1 mg/mL in PBS, 1 mL). The morphology and lattice structure of nanoparticles were observed by transmission electron microscopy (TEM) 39.

### RNA-seq analysis

Thyroid cancer cell line 8305C was treated with AuNP-p-CPS62 and AuNP-CPS62, respectively, and the total RNA of cells was extracted using RNA mini kit (Qiagen, Germany). Enrichment of mRNA, fragmentation, reverse transcription, library construction, Illumina Novaseq 6000 and data analysis were performed by Genergy Biotechnology Co. Ltd. (Shanghai, China). Each experiment was repeated three times.

### *In vitro* anti-tumor efficacy of AuNP-p-CPS62 and AuNP-CPS62

The detailed protocols of cell proliferation, colony formation and cell apoptosis were performed as described previously 40. Half-maximal inhibitory concentration (IC50) values were calculated using the Reed-Muench method.

### *In vivo* anti-tumor efficacy of AuNP-p-CPS62 and AuNP-CPS62

Three- to four-week-old female athymic nude mice were obtained from SLAC laboratory Animal Co., Ltd. To establish xenograft tumor models, the right armpit region of nude mice was subcutaneously inoculated with 5×10^6^ 8305C cells or 3×10^6^ A375 cells. Three days later, we measured tumor sizes every 2 days and calculated tumor volumes using the formula (length × width^2^ × 0.5). When all tumors grew to 50-100 mm^3^, mice bearing tumors derived from 8305C cells and A375 cells were randomly divided into four groups, respectively. AuNP-Ctr, AuNP-p-CPS62 and AuNP-CPS62 were then administered at dose of 1.2 mg/kg every 2 days for 17 days by intraperitoneal injection. The same volume of PBS was used as a control. The dose of peptides in 2 mg/kg solutions was approximately equivalent in weight to 1.5 mg/kg peptides in line with animal ethics. Forty-eight hours after the last dose, mice were anesthetized. Xenograft tumors were isolated and tumor tissues were then embedded in paraffin and sectioned for H&E or IHC staining as described previously 40. Animal studies were approved by Laboratory Animal Center of Xi'an Jiaotong University and all experimental procedures were performed in accordance with institutional guidelines.

### Biosafety evaluation of AuNP-p-CPS62 and AuNP-CPS62

The bio-distribution of AuNP-Ctr, AuNP-p-CPS62 and AuNP-CPS62 in xenograft tumors, and major organs of mice were then detected by Lai analysis testing technology Co., Ltd (Beijing, China). Meanwhile, we monitored the body weights of all mice over the course of treatment and weighed the organs after dissection. Then, we protected the organs and tumors tissues from light for Ex vivo fluorescence imaging. Also, we collected serum samples to measure the levels of CK, ALT, AST, CRE and BUN using specific ELISA kits (#A032-1-1, #C009-2-1, #C010-2-1, #C011-2-1 and #C013-2-1, Jiancheng Bioengineering Institute, Nanjing, China) according to the manufacturer's instructions. Additionally, we collected blood to evaluate CBC including RBC, HGB, PLT, WBC, lymphocyte and eosinophils counts according to standard clinical laboratory procedure. Finally, the tissue sections were stained with H&E.

### Statistical analysis

The correlation analysis between genes was analyzed by linear regression test. Data were compared using the SPSS (package 22.0, Chicago, IL). Data were expressed as the mean ± standard deviation (SD). Statistical significance between groups was determined by two-side unpaired student's t-test or one-way ANOVA. *P* <0.05 was considered to be statistically significant. Unless indicated, the data shown in the figures are representatives.

## Results

### The regulatory relationships among BRAF, TERT and c-Myc

**Figure 1:**
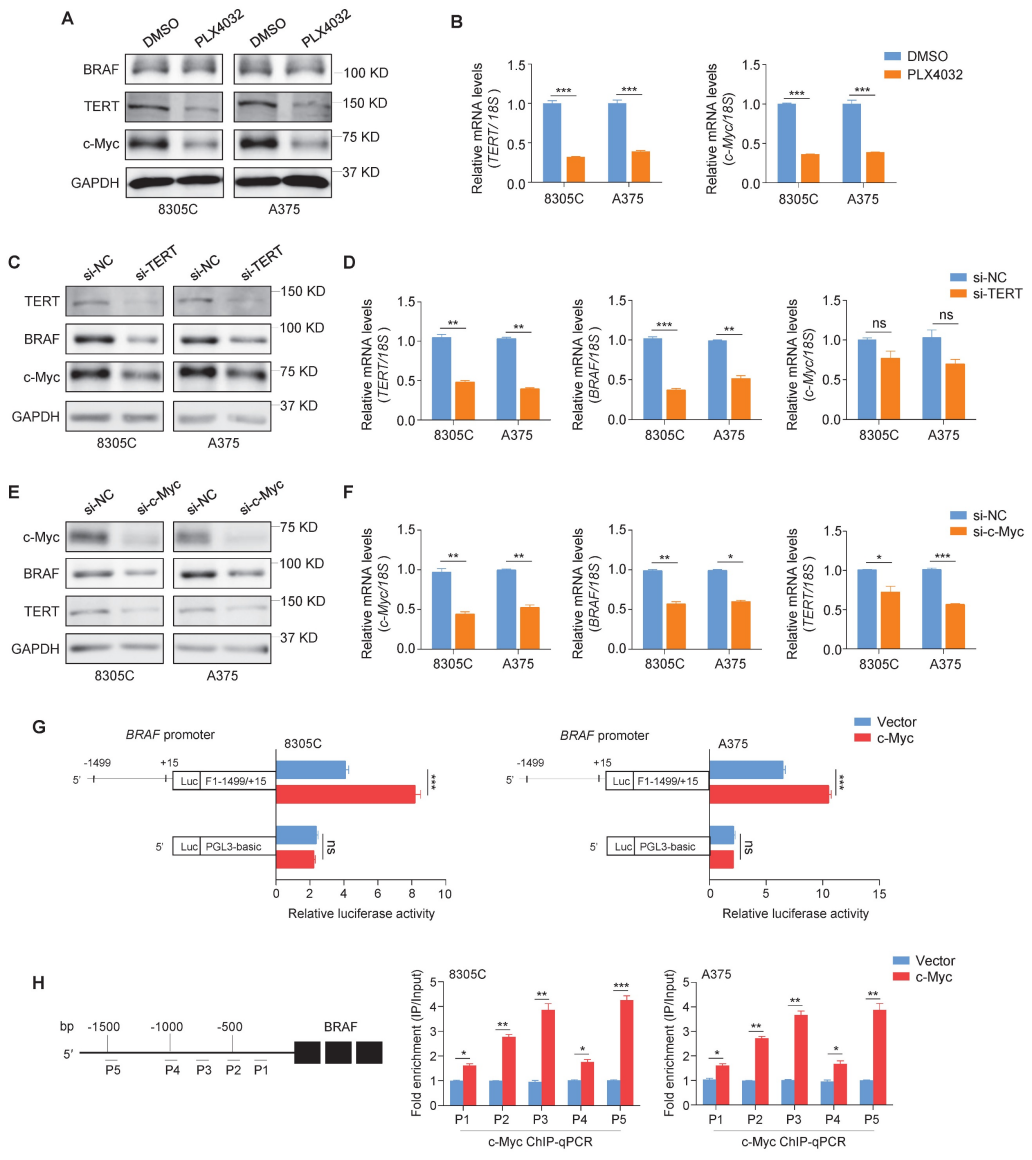
** TERT and c-Myc have mutual internships. (A)** Western blotting analysis was performed to evaluate protein expression of BRAF, TERT and c-Myc upon treating with 4uM BRAF inhibitor PLX4032 in 8305C and A375 cells. GAPDH was used as control. **(B)** qRT-PCR assays were performed to determine the mRNA expression of TERT and c-Myc in 8305C and A375 cells treating with BRAF inhibitor PLX4032. Data were shown as means ± SD. *18S rRNA* was used as a normalized control.** (C)** Upon knocking down of TERT in 8305C and A375 cells by siRNAs, the protein expression of TERT, BRAF and c-Myc were measured by western blotting. GAPDH was used as a loading control.** (D)** The effects of TERT knockdown on TERT, BRAF and c-Myc expression in 8305C and A375 were determined by qRT-PCR assays. *18S rRNA* was used as a normalized control. **(E)** Upon knocking down of c-Myc in 8305C and A375 cells by siRNAs, the protein expression of c-Myc, BRAF and TERT were measured by western blotting. GAPDH was used as a loading control, and the western blotting is representative of three independently preformed experiments. **(F)** The effects of c-Myc knockdown on c-Myc, BRAF and TERT expressions in 8305C and A375 were determined by qRT-PCR assays. *18S rRNA* was used as a normalized control. Data were shown as mean ± SD. Statistical significance between groups was determined by two-side unpaired student's t-test. *,* P* <0.05; **, *P* <0.01; ***, *P* <0.001 (n = 3). **(G)** Dual-luciferase reporter system was used to test the effect of ectopic expression of c-Myc on promoter activity of *BRAF* in 8305C and A375 cells. The empty vector was used as the control, and all the ratios of the Luc/Renilla activity were shown as means ± SD. ***, *P* <0.001 (n = 3). Statistical significance between groups was determined by two-side unpaired student's t-test. **(H)** 8305C and A375 cells expressing c-Myc and control cells were subjected to ChIP-qPCR assays using c-Myc antibody. P1-P5 indicated five regions of BRAF promoter (P1: -582/-436; P2: -718/-562; P3: -980/-869; P4: -1157/-1015; P5: -1499/-1378) (left panel). Fold enrichment was shown as mean ± SD, *,* P* <0.05; **, *P* <0.01; ***, *P* <0.001 (n = 3). Statistical significance between groups was determined by two-side unpaired student's t-test.

To explore the relationships between BRAF, TERT and c-Myc, ATC cell line 8305C and malignant melanoma cell line A375 with co-existence of* BRAF^V600E^* and *pTERT* mutations were selected in this study 41-43. Firstly, we treated these two cell lines with BRAF kinase inhibitor PLX4032, and we found that mRNA and protein levels of TERT and c-Myc were dramatically suppressed upon PLX4032 treatment (Figure 1A and B). Moreover, we knocked down TERT in 8305C and A375 cells, and we found that TERT knockdown dramatically decreased protein levels of BRAF and c-Myc compared with the control (Figure 1C). However, TERT knockdown only decreased mRNA levels of BRAF, while almost did not affect mRNA levels of c-Myc (Figure 1D). We also confirmed this conclusion in glioma cell line SF295 only carrying *pTERT* mutation and colon cancer cell line RKO only carrying *BRAF^V600E^* mutation (Figure S1). In addition, we expectedly observed that knocking down c-Myc in 8305C and A375 cells significantly decreased protein and mRNA levels of BRAF and TERT (Figure 1E and F). These findings suggest that there is a complex regulatory network among BRAF, TERT and c-Myc.

*TERT* has been proved to be a downstream target of c-Myc 21. To validate that *BRAF* is another downstream target of c-Myc, we predicted the presence of potential binding sites of c-Myc in the promoter region of *BRAF* using online tools (http://www.genecards.org and http://jaspar2014.genereg.net). Next, we constructed the luciferase reporter plasmid pGL3-BRAF-Luc (-1499/+15) by cloning the *BRAF* promoter into a pGL3-Basic-Luc luciferase plasmid. After co-transfecting pGL3-BRAF-Luc or pGL3-Basic-Luc with luciferase expressing plasmids (pRL-TK) into 8305C and A375 cells, we found that ectopic expression of c-Myc significantly enhanced the promoter activity of *BRAF* compared with the control (Figure 1G). To determine whether c-Myc regulates the transcription of *BRAF* by directly binding to its promoter, ChIP assays were performed in c-Myc overexpressing 8305C and A375 cells and control cells, followed by qPCR assay using primers to specifically amplify five different regions within *BRAF* promoter. The results showed that all five regions within *BRAF* promoter were significantly enriched in c-Myc overexpressing cells compared with control cells, especially P2, P3 and P5 regions, which were enriched by more than 2-fold (Figure 1H). These results indicate that *BRAF* is a direct downstream target of c-Myc. Meanwhile, we ectopically expressed c-Myc in TERT-knockdown 8305C and A375 cells. The results showed that ectopic expression of c-Myc reversed the inhibitory effect of TERT knockdown on BRAF expression (Figure S2), indicating that the regulatory effect of TERT on BRAF is mediated by c-Myc. Taken together, our data indicates that there exists a reciprocal regulatory role among BRAF, TERT and c-Myc.

### TERT interacts with and stabilizes c-Myc

**Figure 2:**
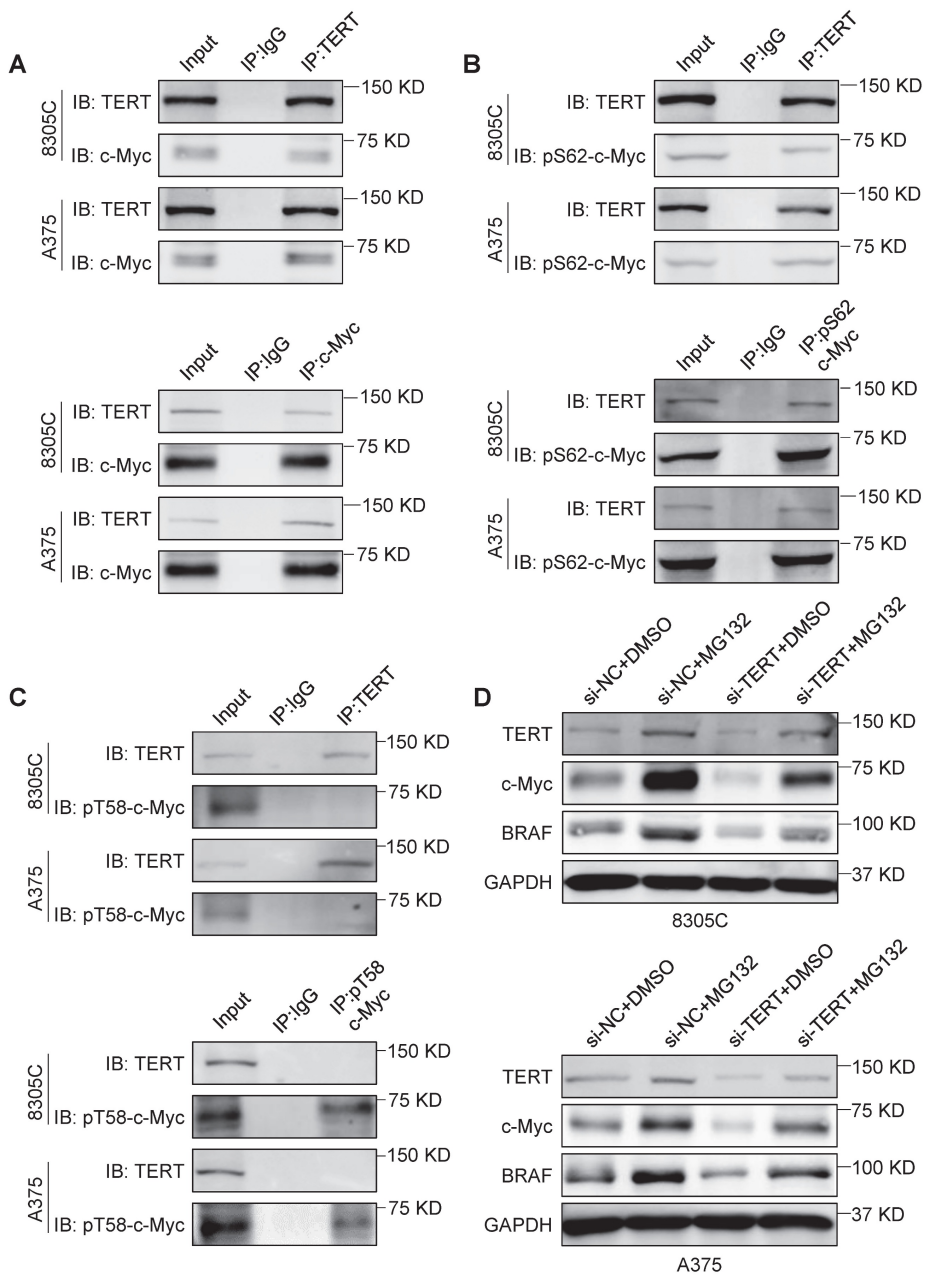
** TERT is able to stabilize c-Myc. (A)** Reciprocal Co-IP assays were performed in 8305C and A375 cells to determine the interaction between TERT and c-Myc using the indicated antibodies. **(B)** Reciprocal Co-IP assays were performed in 8305C and A375 cells to determine the interaction between TERT and pS62-c-Myc using the indicated antibodies.** (C)** Reciprocal Co-IP assays were performed in 8305C and A375 cells to determine the interaction between TERT and pT58c-Myc using the indicated antibodies. The antibody IgG was used as negative control, and the co-immunoprecipitation is representative of three independently preformed experiments. **(D)** 8305C and A375 cells were pretreated with 25 μM MG132 or DMSO for four hours and treated with si-TERT. The western blotting analysis was then used to evaluate TERT, BRAF and c-Myc expression. GAPDH was used as control.

A previous study indicated that TERT could stabilize c-Myc by directly binding to its MBI domain 32. However, the exact mechanism remains unclear. In this study, we further validated that TERT interacted with c-Myc in 8305C, A375, SF295 and RKO cells by Co-IP assays (Figure 2A and Figure S3A). The MBI domain of c-Myc contains two crucial phosphorylation sites, Ser62 and Thr58 28. Phosphorylation of c-Myc at Ser62 (pS62) is required for its stability, while phosphorylation of c-Myc at Thr-58 (pT58) is necessary for its degradation 28. We confirmed that TERT could directly bind to pS62-c-Myc but not pT58-c-Myc in 8305C, A375, SF295 and RKO cells by Co-IP assays (Figure 2B and C; Figure S3B and C). Next, we treated these cells with proteasome inhibitor MG132, and found that MG132 could reverse the inhibitory effect of TERT knockdown on the expression of c-Myc and BRAF (Figure 2D and Figure S3D). These data, taken together, indicate that TERT interacts with pS62-c-Myc to prevent its ubiquitin/proteasome-mediated degradation.

### c-Myc negatively regulates the transcription of *PP2Ac*

**Figure 3:**
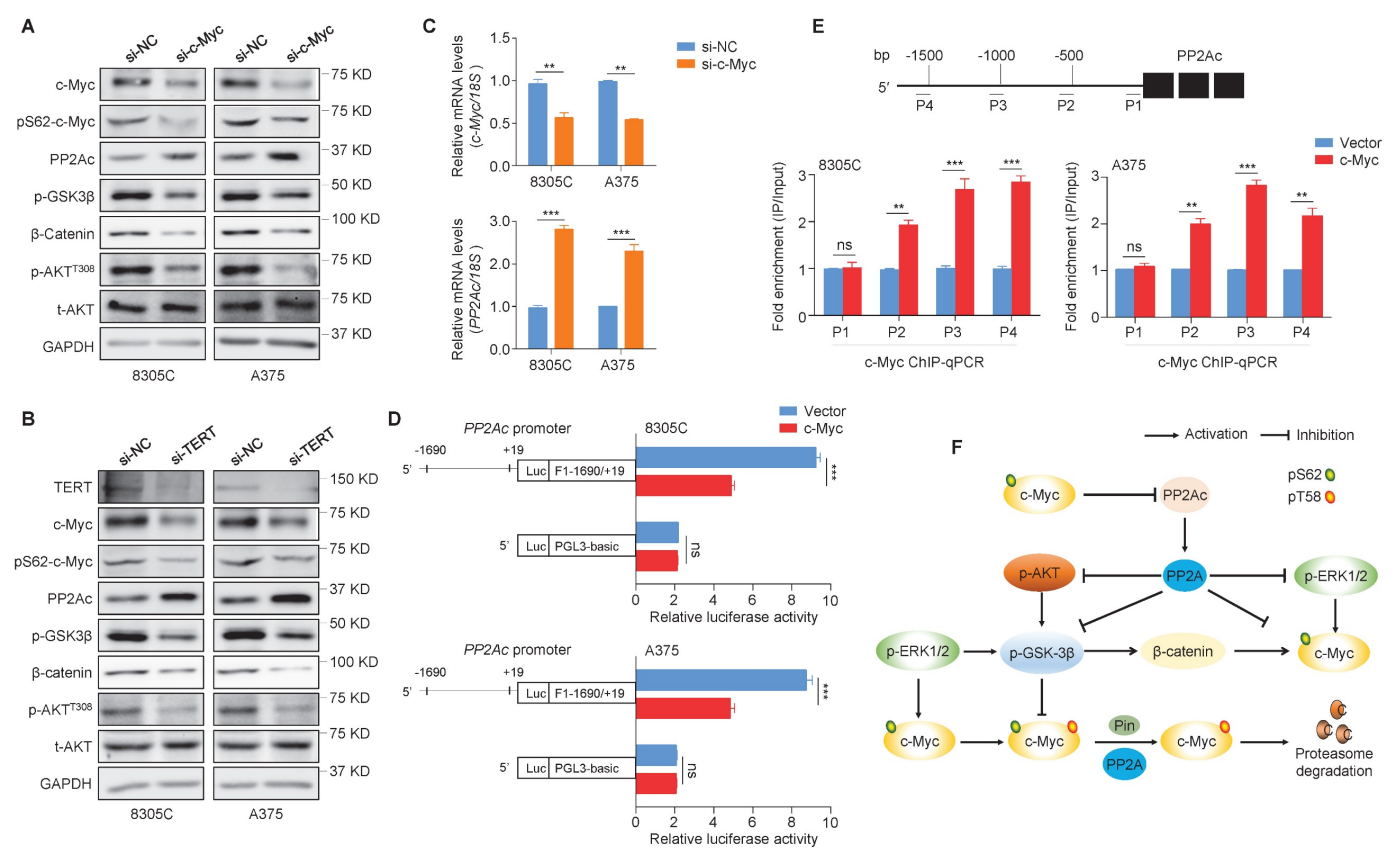
** c-Myc transcriptionally inhibits *PP2Ac* expression. (A)** The effects of c-Myc knockdown on the expression of pS62-c-Myc, PP2Ac, p-GSK3β, β-catenin and p-AKT^T308^ in 8305C and A375 cells were determined by Western blotting assays. **(B)** The TERT knockdown on the expression of c-Myc, pS62-c-Myc, PP2Ac, p-GSK3β, β-catenin and p-AKT^T308^ in 8305C and A375 cells were determined by Western blotting assays. GAPDH was used as a loading control and the western blotting is representative of three independently preformed experiments. **(C)** The effects of c-Myc knockdown on PP2Ac expression in 8305C and A375 were determined by qRT-PCR assays. *18S rRNA* was used as a normalized control. Data were shown as mean ± SD. **, *P* <0.01; ***, *P* <0.001 (n = 3). Results with statistical significance were obtained through the use of two-side unpaired Student's t-tests. **(D)** Dual-Luciferase Reporter assay system was used to test the function of ectopic expression of c-Myc on promoter activity of *PP2Ac* in 8305C and A375 cells. The empty vector was used as the control, and all the ratio of the Luc/Renilla activity were shown as means ± SD. ***, *P* <0.001 (n = 3). Statistical significance between groups was determined by two-side unpaired Student's t-test. **(E)** 8305C and A375 cells expressing c-Myc and control cells were subjected to ChIP-qPCR assays using c-Myc antibody. P1-P4 indicated four different regions of PP2Ac promoter (P1: -143/0; P2: -786/-642; P3: -1161/-941; P4: -1689/-1489) (upper panel). Fold enrichment was shown as mean ± SD, **, *P* <0.01; ***, *P* <0.001 (n = 3). Statistical significance between groups was determined by two-side unpaired Student's t-test.** (F)** c-Myc can transcriptionally inhibits the transcription of *PP2Ac* to suppress the dephosphorylation activity of PP2A on p-ERK, p-AKT^T308^, and p-GSK3β, protecting c-Myc from ubiquitination degradation pathway.

The activation of MEK/ERK signaling pathway by *BRAF^V600E^* mutation mediates S62 phosphorylation of c-Myc to maintain its stability, while GSK3β phosphorylates c-Myc at T58 together with PP2A dephosphorylates c-Myc at S62, leading to its ubiquitylation and subsequent degradation 28. The phosphorylation of AKT and ERK triggers GSK3β deactivation via its phosphorylation at Ser9 to cause the accumulation of β-catenin, which is a transcriptional activator of c-Myc 44. We knocked down c-Myc in 8305C and A375 cells, and found that c-Myc knockdown caused a significant increase in protein levels of PP2Ac (the core catalytic subunit of PP2A), while lead to a dramatic decrease in protein levels of pS62-c-Myc, phosphorylated GSK3β (p-GSK3β), β-catenin and phosphorylated AKT at Thr308 (p-AKT^T308^) compared with the control (Figure 3A). This indicates that c-Myc can maintain its own stability by suppressing ubiquitination-dependent degradation pathway. Also, we found that knocking down TERT and BRAF in 8305C and A375 cells had a similar effect on PP2Ac, pS62-c-Myc, p-GSK3β, β-catenin and p-AKT^T308^ with c-Myc knockdown (Figure 3B and Figure S4).

The strong protein phosphatase 2A (PP2A) can regulate the activity of MAPK/ERK, PI3K/AKT, Wnt, mTOR and many other signaling pathways via dephosphorylation of over 300 substrates 45. To determine the mechanism by which c-Myc regulates PP2Ac, we performed qRT-PCR assays in 8305C and A375 cells, showing that c-Myc knockdown significantly increased mRNA expression of* PP2Ac* compared with the control (Figure 3C). Thus, we suppose that *PP2Ac* may be a downstream target of c-Myc. Firstly, we used online tools (http://www.genecards.org and http://jaspar2014.genereg.net) to predict the presence of potential binding sites of c-Myc in *PP2Ac* promoter. Next, we proved that c-Myc regulated the transcription of *PP2Ac* by directly binding to its promoter using the dual-luciferase reporter assay (Figure 3D) and ChIP-qPCR assay (Figure 3E). Collectively, our data suggest that *PP2Ac* is negatively regulated by c-Myc at the transcriptional levels, which in turn reduces the dephosphorylation effect of PP2A on pS62-c-Myc, p-GSK3β, p-AKT^T308^ and p-ERK, thus enhancing protein stability of c-Myc (Figure 3F).

### Peptide-gold nanoparticles induce the ubiquitin-mediated degradation of c-Myc by blocking the interaction between TERT and c-Myc

**Figure 4:**
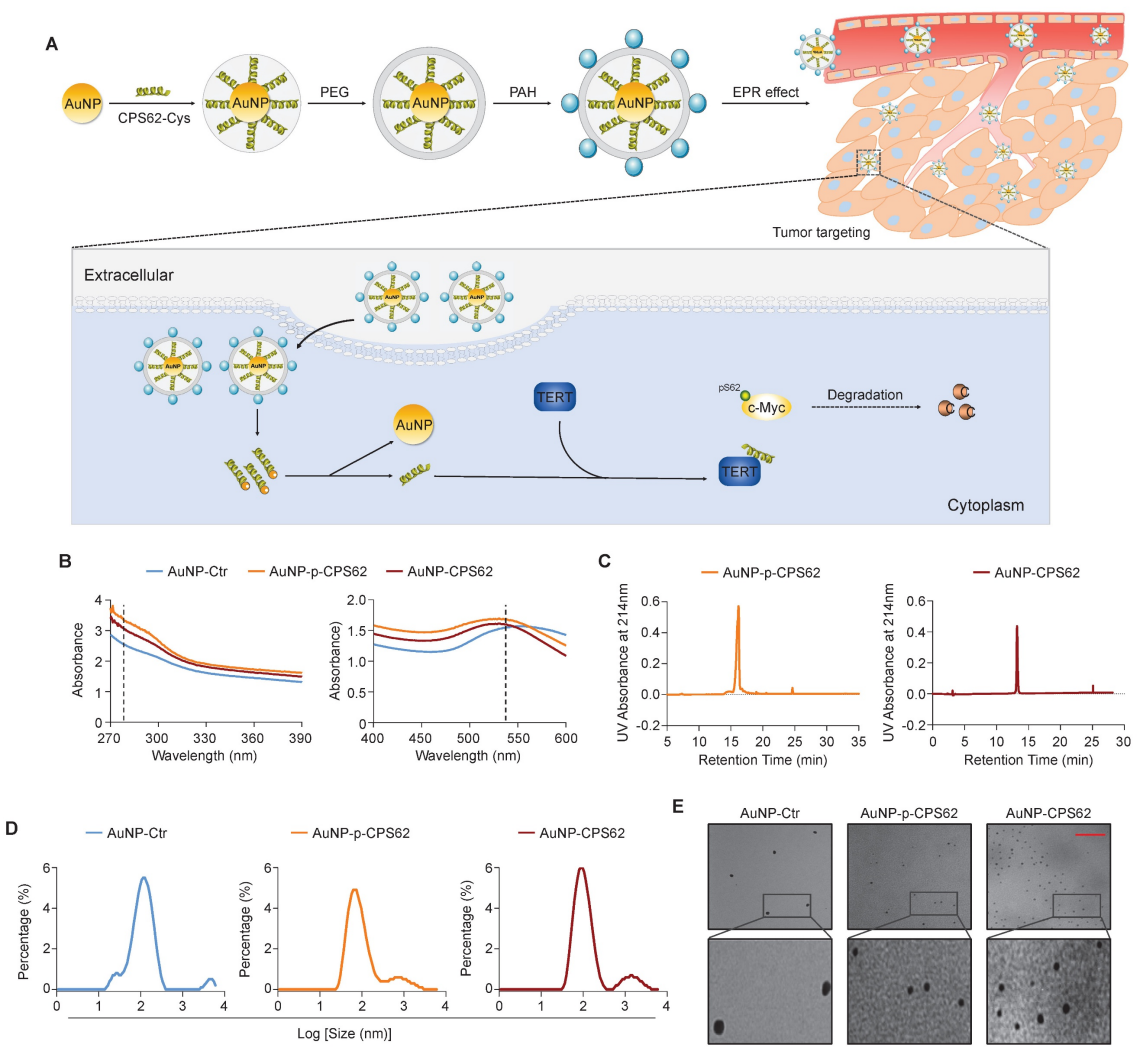
** Synthesis and physicochemical properties of polypeptides**-**gold nanoparticles. (A)** Schematic depiction of the synthesis of polypeptides-gold nanoparticles. **(B)** Ultraviolet-visible spectra of AuNP-Ctr, AuNP-p-CPS62 and AuNP-CPS62 were measured in HEPES buffer (pH 7.4) at the concentration of 0.2 mg/ml. The peaks of three curves at ~280 nm were the characteristic absorption peak of peptides bond. The peaks of three curves at ~540 nm were the plasma resonance peak of gold nanoparticles.** (C)** Redox-dependent release was characterized in chemically synthesized AuNP-p-CPS62 and AuNP-CPS62 by RP-HPLC.** (D)** Nanoparticles particle size distribution diagram. The Y-axis represents the percentage of each particle size degree. The X-axis is the particle size. **(E)** Upper images are representative TEM image of AuNP-Ctr, AuNP-p-CPS62 and AuNP-CPS62 at pH 7.4. The lower images show magnifications of the area indicated by the black squares. Scale bars, 1000 nm.

Our data indicated that c-Myc plays a central role in the mutual regulation of *BRAF^V600E^* and *pTERT* mutations and that the interaction between c-Myc and TERT is essential for maintaining protein stability of c-Myc. Thus, we designed two peptides to block the interaction between pS62-c-Myc and TERT, each consisting of 20 amino acids in the c-Myc/TERT interaction domain, characterized by an N-terminal acetylation, a non-phosphorylated threonine at position 58, a C-terminal cysteine and either a phosphorylated or an unphosphorylated serine at position 62 (pS62 and S62). Considering that the peptides have poor *in vivo* stability and weak membrane penetrating ability 46, and gold nanoparticles (AuNPs) are usually used as an efficient drug delivery system including peptides 35, we thus constructed corresponding peptide-gold nanoparticles (AuNP-p-CPS62 and AuNP-CPS62) to block the interaction between c-Myc and TERT. The preparation and working principle of peptide-gold nanoparticles were illustrated in Figure 4A. To assess the conjugation efficiency of peptides with AuNPs, we measured the absorbance of peptide-gold nanoparticles using UV-vis spectroscopy. The peaks of AuNP-p-CPS62 and AuNP-CPS62 curves at approximately 280 nm and 540 nm were higher than control AuNP (AuNP-Ctrl) (Figure 4B). Moreover, the release peak times for AuNP-p-CPS62 and AuNP-CPS62 were around 15 min by redox-dependent release and HPLC (Figure 4C), meaning that conjugated peptide-gold nanoparticles may be tremendously pure in the solution. The particle sizes of peptide-gold nanoparticles were then determined by Nano-ZS (Figure 4D) and observed by transmission electron microscopy (TEM) (Figure 4E), showing that the particle size of AuNP-Ctr was 115.1 ± 4.66 nm, AuNP-p-CPS62 was 85.9 ± 2.81 nm and AuNP-CPS62 was 106.68 ± 2.23 nm. These findings indicate the successful conjugation of peptides with AuNPs and the nanoparticle sizes are suitable for crossing the cell membrane.

**Figure 5:**
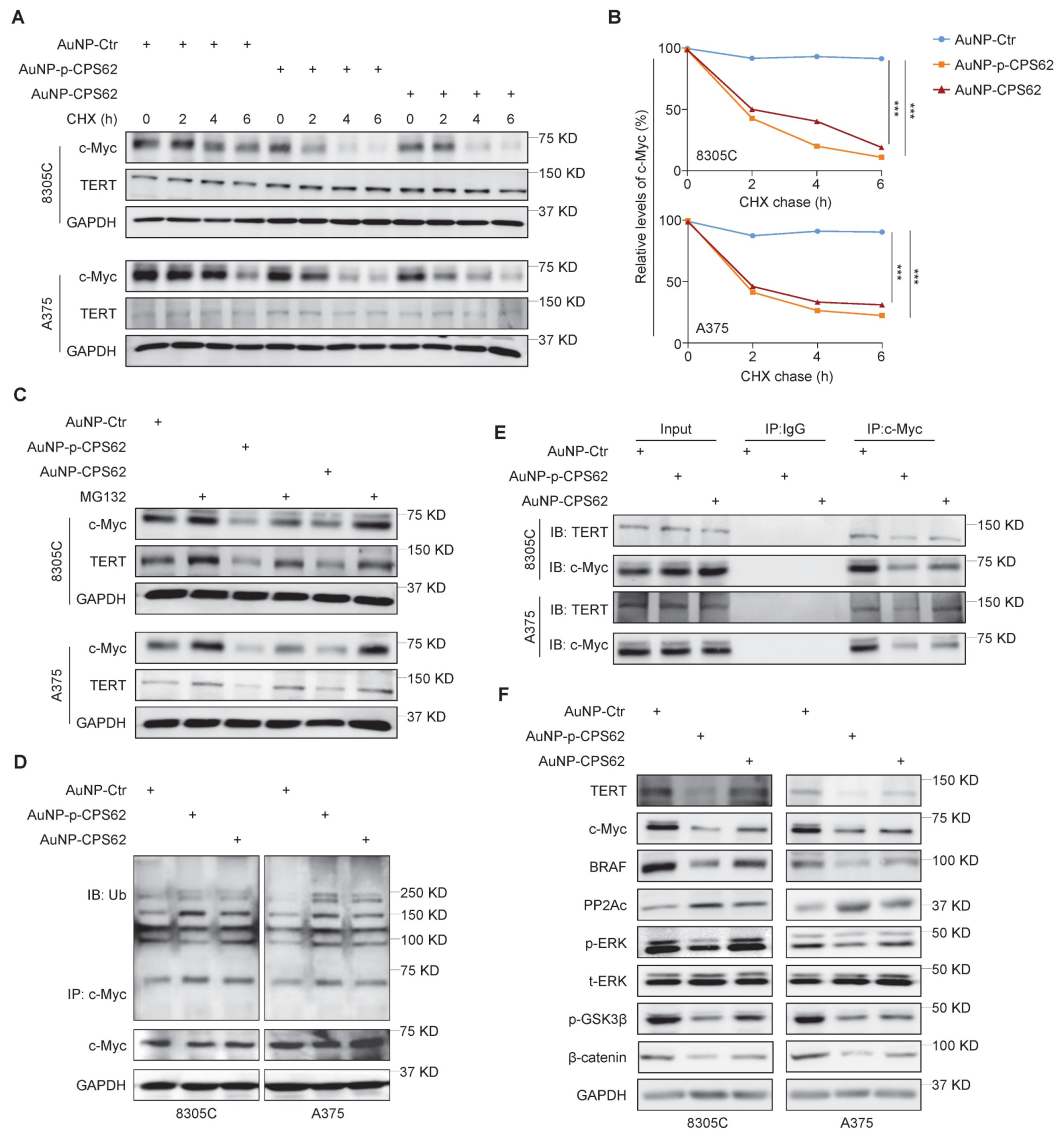
** AuNP-p-CPS62 and AuNP-CPS62 promote c-Myc degradation. (A)** 8305C and A375 cells which pretreated with 200 µg/ml cycloheximide at the indicated times were treated with 30 nM AuNP-Ctr, 16 nM AuNP-p-CPS62 or 23 nM AuNP-CPS62 for 24h. Cell lysates were then prepared and immunoblotted for the indicated proteins (left panels). **(B)** The band intensity of c-Myc in cycloheximide-treated cells was first normalized to GAPDH levels and then further normalized to the intensity of DMSO-treated cells (right panels). Data were shown as mean ± SD. ***, *P* <0.001 (n = 3). Statistical significance between groups was determined by one-way ANOVA. **(C)** 8305C and A375 cells which pretreated with 25 µM MG132 or DMSO for 4 h and were treated with 30 nM AuNP-Ctr, 16 nM AuNP-p-CPS62 or 23 nM AuNP-CPS62 48h and then subjected to western blotting analysis using the indicated antibodies. GAPDH was used as a loading control. **(D)** 8305C and A375 cells pretreated with AuNP-Ctr, AuNP-p-CPS62 or AuNP-CPS62 were treated with 25 µM MG132 for 4 h. Lysates were then mixed with anti-c-Myc antibody and conjugated with agarose. Next, ubiquitination levels of c-Myc were determined by immunoblotting using an anti-ubiquitin (Ub) antibody. Input samples were taken prior to the above steps. **(E)** Cell lysates of 8305C and A375 cells treating with AuNP-Ctr, AuNP-p-CPS62 or AuNP-CPS62 were immunoprecipitated with anti-TERT antibody, and the precipitated proteins were subjected to western blotting analysis using anti-c-Myc antibody. The antibody IgG was used as a negative control and the co-immunoprecipitation is representative of three independently preformed experiments. **(F)** Western blotting analysis was used to evaluate protein expression of TERT, c-Myc, BRAF, PP2Ac, p-ERK, p-GSK3β and β-catenin in 8305C and A375 cells treated with AuNP-Ctr, AuNP-p-CPS62 or AuNP-CPS62. Using GAPDH as a loading control and the western blotting is representative of three independently preformed experiments.

We next used cycloheximide (CHX) to block *de novo* protein synthesis and then evaluated its effect on c-Myc protein stability in AuNP-p-CPS62- or AuNP-CPS62-treated 8305C and A375 cells and their control cells. The results showed that AuNP-p-CPS62 and AuNP-CPS62 markedly accelerated c-Myc degradation in 8305C and A375 cells, with little effect on the stability of TERT proteins (Figure 5A and B). This effect could be reversed by MG132 pretreatment (Figure 5C). These findings indicate that AuNP-p-CPS62 or AuNP-CPS62 induces the ubiquitin-mediated degradation of c-Myc. Moreover, we performed Co-IP combined with western blotting assays to examine the ubiquitination levels of c-Myc proteins in 8305C and A375 cells after treatment with AuNP-p-CPS62 or AuNP-CPS62. The results showed that AuNP-p-CPS62 or AuNP-CPS62 obviously increased the ubiquitination levels of c-Myc proteins compared with the control (Figure 5D). We also investigated the effect of AuNP-p-CPS62 or AuNP-CPS62 on the interaction between c-Myc and TERT using Co-IP assays, and we demonstrated that AuNP-p-CPS62 and AuNP-CPS62, especially the former, substantially weakened the interaction between c-Myc and TERT (Figure 5E). We also evaluated the impact of AuNP-p-CPS62 or AuNP-CPS62 on the expression of crucial proteins involved in c-Myc degradation in 8305C and A375 cells using western blotting analysis. The results showed that AuNP-p-CPS62 and AuNP-CPS62, particularly the former, noticeably down-regulated the levels of TERT, c-Myc, BRAF, p-ERK, p-GSK3β and β-catenin, while markedly increased the levels of PP2Ac (Figure 5F). These results indicate that AuNP-p-CPS62 and AuNP-CPS62, especially the former, promote c-Myc degradation by blocking the interaction between c-Myc and TERT.

Next, we expectedly observed that AuNP-p-CPS62 and AuNP-CPS62 caused a significant decrease in mRNA levels of *TERT* and *BRAF* and an increase in mRNA levels of *PP2Ac* (Figure S5). Additionally, we performed the RNA-seq to define the impact of AuNP-p-CPS62 and AuNP-CPS62 treatment on global gene expression profiles. The results showed that AuNP-p-CPS62 and AuNP-CPS62 treatment caused a significant change in gene expression profiles compared with the control (Figure S6A and Supplementary Table 1). There were consistent 176 down-regulated differentially expressed genes (DEGs) and the shared 300 up-regulated DEGs upon AuNP-p-CPS62 and AuNP-CPS62 treatment (Supplementary Table 2 and Supplementary Table 3). Of them, we expectedly observed that *BRAF* and *TERT* were down-regulated and *PP2Ac* was up-regulated in AuNP-p-CPS62 and AuNP-CPS62 treated cells. By analyzing the SangerBox database, we found that the above DEGs caused by AuNP-p-CPS62 and AuNP-CPS62 treatment were mainly involved in multiple cancer-related pathways (Figure S6B). The circle diagram partially showed that these DEGs were enriched in different pathways (Figure S6C). Importantly, some of these DEGs have been identified as the downstream targets of c-Myc, such as *TERT*, *CDKN1A*, *CDH3*, *CCNA2*, *PGK1*, *NDRG1* and *ASS1*. These data, collectively, suggest that AuNP-p-CPS62 or AuNP-CPS62 treatment can cause a change in global gene expression profiles in cancer cells, including the downstream targets of c-Myc, meanwhile, may exert their anti-tumor effects by regulating the transcription of other tumor-related genes.

### Peptide-gold nanoparticles inhibit the* in vitro* growth of *BRAF^V600E^/pTERT* double mutated cancer cells

**Figure 6:**
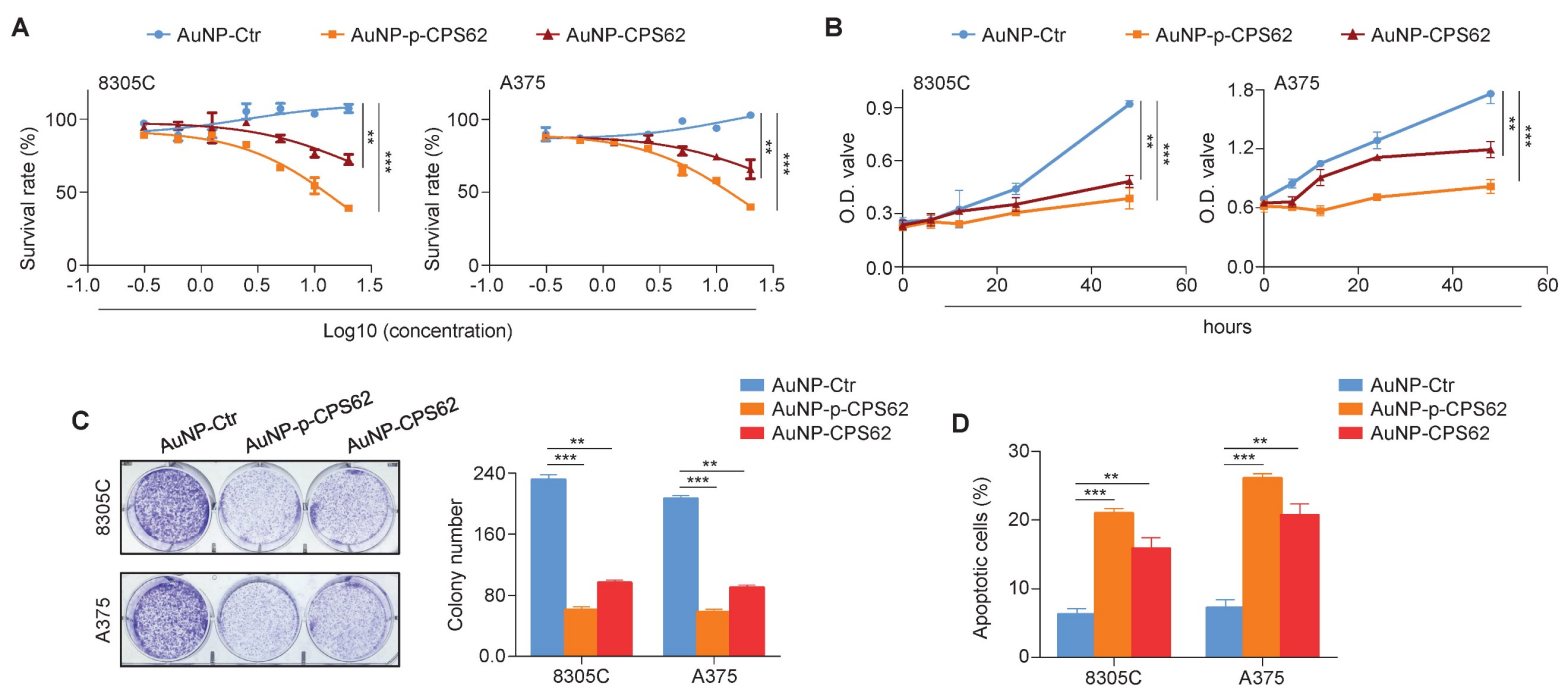
** Biological function of polypeptides**-**gold nanoparticles on *BRAF^V600E^/pTERT* double -mutant cells *in vitro*. (A)** The indicated cell lines were treated with varying concentrations of AuNP-Ctr, AuNP-p-CPS62 or AuNP-CPS62 for 72 hours. The MTT assay was then performed to evaluate cell proliferation, and Reed-Muench method was applied to calculate the IC_50_ values. Data were presented as mean ± SD (n=5). **(B)** Cell viability upon treatment with 30 nM AuNP-Ctr, 16 nM AuNP-p-CPS62 or 23 nM AuNP-CPS62 were determined by the MTT assay. Data were shown as mean ± SD. **, *P* <0.01; ***, *P* <0.001 (n=5). Statistical significance between groups was determined by one-way ANOVA. **(C)** Cells were treated with 30 nM AuNP-Ctr, 16 nM AuNP-p-CPS62 or 23 nM AuNP-CPS62 for 10 days, and stained with crystal violet. The left panels show representative images of colony formation. Quantitative analysis of colony numbers is shown in the right panes. Data were presented as mean ± SD. **, *P* <0.01; ***, *P* <0.001 (n = 3). Statistical significance between groups was determined by one-way ANOVA.** (D)** 8305C and A375 cells were treated with 30 nM AuNP-Ctr, 16 nM AuNP-p-CPS62 or 23 nM AuNP-CPS62 for 72 h, and cell apoptosis was evaluated by Annexin V-PI staining and flow cytometric analysis. The data were presented as mean ± SD. **, *P* <0.01; ***, *P* <0.001 (n = 3). Statistical significance between groups was determined by one-way ANOVA.

We next investigated the *in vitro* anti-tumor efficacy of AuNP-p-CPS62 and AuNP-CPS62 in 8305C and A375 cells. Firstly, these two cell lines were treated with incremental concentrations of peptide-gold nanoparticles from 0.625 to 40 nM for 72 h, and their IC_50_ values were then measured (Table S8). The results showed that AuNP-p-CPS62 effectively inhibited cancer cell growth than AuNP-CPS62, while AuNP-Ctr had minimal impact on cell growth (Figure 6A). This was further supported by MTT (Figure 6B) and colony formation (Figure 6C) assays. We also assessed cell apoptosis after a 72-h treatment of peptide-gold nanoparticles, and we found that AuNP-p-CPS62 and AuNP-CPS62 induced more significant apoptotic cells compared with AuNP-Ctr, especially the former (Figure 6D). This was also verified in *BRAF^V600E^/pTERT* double mutated cancer cells M14 and BCPAP (Figure S7). These findings indicate that AuNP-p-CPS62 and AuNP-CPS62, particularly AuNP-p-CPS62, can effectively inhibit the *in vitro* growth of *BRAF^V600E^/pTERT* double mutated cancer cells.

### Peptide-gold nanoparticles inhibit the* in vivo* growth of *BRAF^V600E^/pTERT* double mutated cancer cells

**Figure 7:**
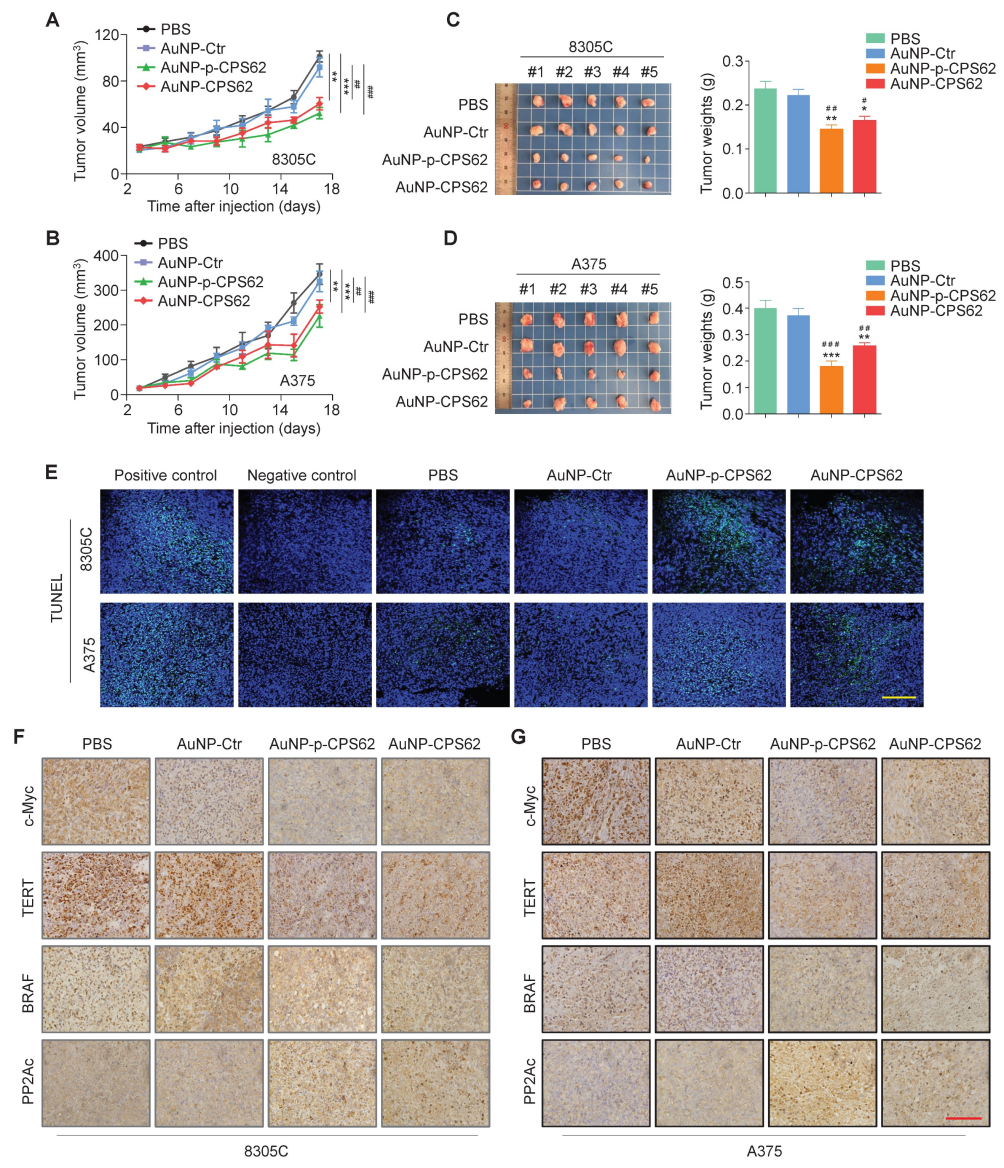
** Inhibition of xenograft tumor growth by AuNP-p-CPS62 and AuNP-CPS62. (A and B)** Xenograft tumor models were established by subcutaneous inoculation of 8305C or A375 cells, and mice were then randomized into four different groups (PBS, AuNP-Ctr, AuNP-p-CPS62 and AuNP-CPS62; n = 5/group), respectively. The panels show time courses of tumor growth in mice with the indicated treatments. Day 0 indicates the time point of cancer cell injection. Data were shown as mean ± SD. The symbol “^#^” indicates the significant difference between PBS group and AuNP-Ctr, AuNP-p-CPS62 or AuNP-CPS62 groups. The symbol “*” indicates the significant difference between AuNP-Ctr group and AuNP-p-CPS62 or AuNP-CPS62 groups. ^##^, *P* < 0.01; ^###^,* P* < 0.001. **, *P* < 0.01; ***,* P* < 0.001. Statistical significance between groups was assessed using one-way ANOVA. **(C and D)** Images of dissected tumors from nude mice are presented in the left panels. Histogram represents the average weight of xenograft tumors from AuNP treatment and control groups (right panels). ^#^, *P* < 0.05; ^##^, *P* < 0.01; ^###^,* P* < 0.001. *, *P* < 0.05; **, *P* < 0.01; ***,* P* < 0.001 (n=5/group). Statistical significance between groups was assessed using a two-sided unpaired Student's t-test.** (E)** Induction of apoptosis and cell death in xenograft tumors from control and AuNP-treated mice were shown by the TUNEL assay. Green represents target TUNEL staining, and blue represents Hoechst33342 staining for nuclei. Scale bars, 50 µm.** (F and G)** Representative xenograft tumor sections from polypeptides-gold nanoparticles treated and corresponding control groups were subjected to IHC staining. Scale bars: 200 µm.

To determine the *in vivo* anti-tumor activity of peptide-gold nanoparticles, we established xenograft mouse models by subcutaneously inoculating 8305C and A375 cells. These mice were then randomly divided into four groups: PBS, AuNP-Ctr, AuNP-p-CPS62 and AuNP-CPS62. The results showed that 8305C cell- and A375 cell-derived xenograft tumors treated with AuNP-p-CPS62 and AuNP-CPS62 grew slower (Figure 7A and B) and weighed less (Figure 7C and D) compared with AuNP-Ctrl- or PBS-treated groups, while AuNP-Ctr-treated tumors exhibited no changes relative to PBS-tumors (Figure 7A-D). Next, we performed IHC assays to evaluate the levels of Ki-67 in xenograft tumors, and we found that the percentage of Ki-67-positive cells was significantly lower in the tumors from mice treated with AuNP-p-CPS62 or AuNP-CPS62 compared with those treated with AuNP-Ctr or PBS (Figure S8). TUNEL assays were then performed to evaluate cell apoptosis in xenograft tumors. The results showed that there were more apoptotic cells in AuNP-p-CPS62- and AuNP-CPS62-treated tumors, while fewer apoptotic cells were observed in the PBS- and AuNP-Ctr-treated tumors (Figure 7E and Figure S9). Overall, it seemed that the anti-tumor efficacy of AuNP-p-CPS62 was better than AuNP-CPS62, further supporting the conclusions of *in vitro* experiments described above. We next examined the protein levels of c-Myc, TERT, BRAF and PP2Ac in tumor tissues using IHC assays. The results showed that the expressions of BRAF, TERT, and c-Myc were significantly decreased in the tumors from mice treated with AuNP-p-CPS62 and AuNP-CPS62 compared with those treated with AuNP-Ctr or PBS, while the expression of PP2Ac was markedly increased (Figure 7F and G; Figure S10). Our data provides compelling evidence that peptide-gold nanoparticles, particularly AuNP-p-CPS62, possess potent anti-cancer ability by down-regulating the expression of c-Myc, TERT, and BRAF via blockade of c-Myc/TERT interaction.

**Figure 8:**
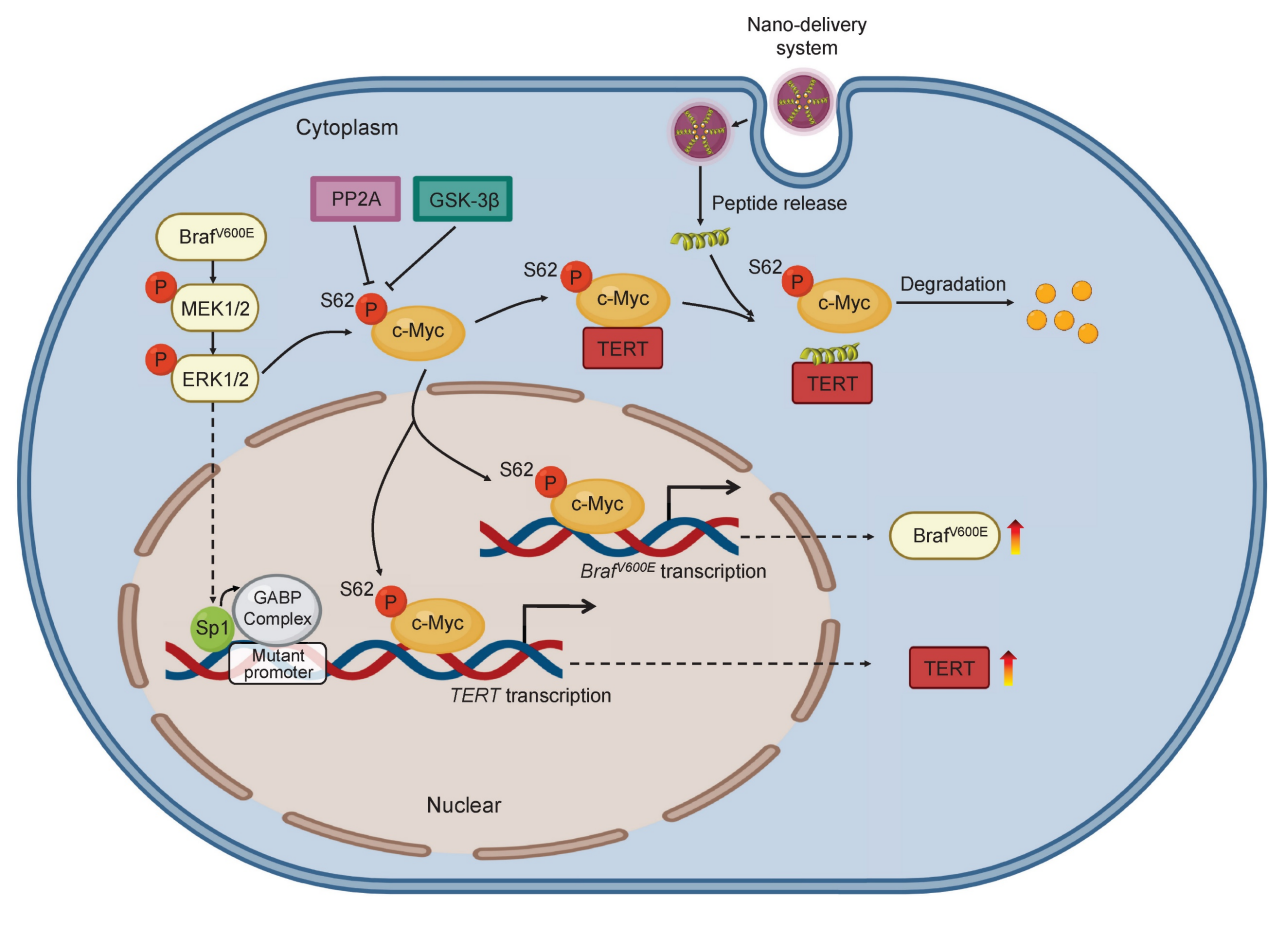
** A schematic model for polypeptides**-**gold nanoparticles targeting c-Myc and inhibiting malignant tumor progression.** In *BRAF* and *TERT* dual-mutant tumors, BRAF can phosphorylate ERK to transcriptionally activate TERT by promoting Sp1 phosphorylation or GABP complex formation. Additionally, the transcription factor c-Myc protein is stabilized in three ways. Firstly, c-Myc can transcriptionally active BRAF to trigger hyperactivated ERK signaling which in turn phosphorylate Ser62 to stabilize c-Myc. Secondly, TERT which is transcribed by c-Myc can tightly combine with pS62-c-Myc, protecting c-Myc from degradation through inhibiting dephosphorylation at Ser62 by PP2A. Therefore, BRAF can also be indirectly positively regulated by TERT. Thirdly, c-Myc can negatively regulate the transcription of PP2Ac to suppress the activity of PP2A, preventing c-Myc ubiquitination degradation pathway. Conclusively, there are positive feedback loops between BRAF, TERT and c-Myc in cancer cells for enhancing c-Myc stabilization in cascade. The exogenous polypeptides-gold nanoparticles are designed to compete with pS62-c-Myc on targeted binding with TERT, and polypeptides-gold nanoparticles possess potent anticancer ability through speeding c-Myc degradation and breaking c-Myc related feedback loops in *BRAF* and *TERT* dual-mutant tumor cells.

Based on the above findings, we propose a model to illustrate the mechanism by which blocking the interaction between c-Myc and TERT kills cancer cells with co-existence of* BRAF^V600E^* and *pTERT* mutations (Figure 8). Briefly, in *BRAF^V600E^/pTERT* double mutated cancer cells, *BRAF^V600E^* mutation-mediated activation of MAPK/ERK signaling pathway phosphorylates c-Myc at Ser62 to protect it from degradation. Meanwhile, activated ERK also phosphorylates Sp1 to facilitate the formation of GABP complex, thereby enhancing *TERT* transcription. TERT interacts with pS62-c-Myc to prevent its ubiquitin/proteasome-mediated degradation. In turn, c-Myc promotes the transcription of *BRAF* and *TERT*. In addition, c-Myc negatively regulates the transcription of *PP2Ac* to inhibit its dephosphorylation of pS62-c-Myc, guaranteeing the protein stability of c-Myc. Thus, there exists a complex regulatory network among BRAF, TERT and c-Myc. AuNP-p-CPS62 and AuNP-CPS62, especially the former, disrupt the above regulatory network by blocking interaction between TERT and c-Myc to promote c-Myc degradation, thereby effectively killing *BRAF^V600E^/pTERT* double mutated tumors.

### Safety evaluation of peptide-gold nanoparticles

**Figure 9:**
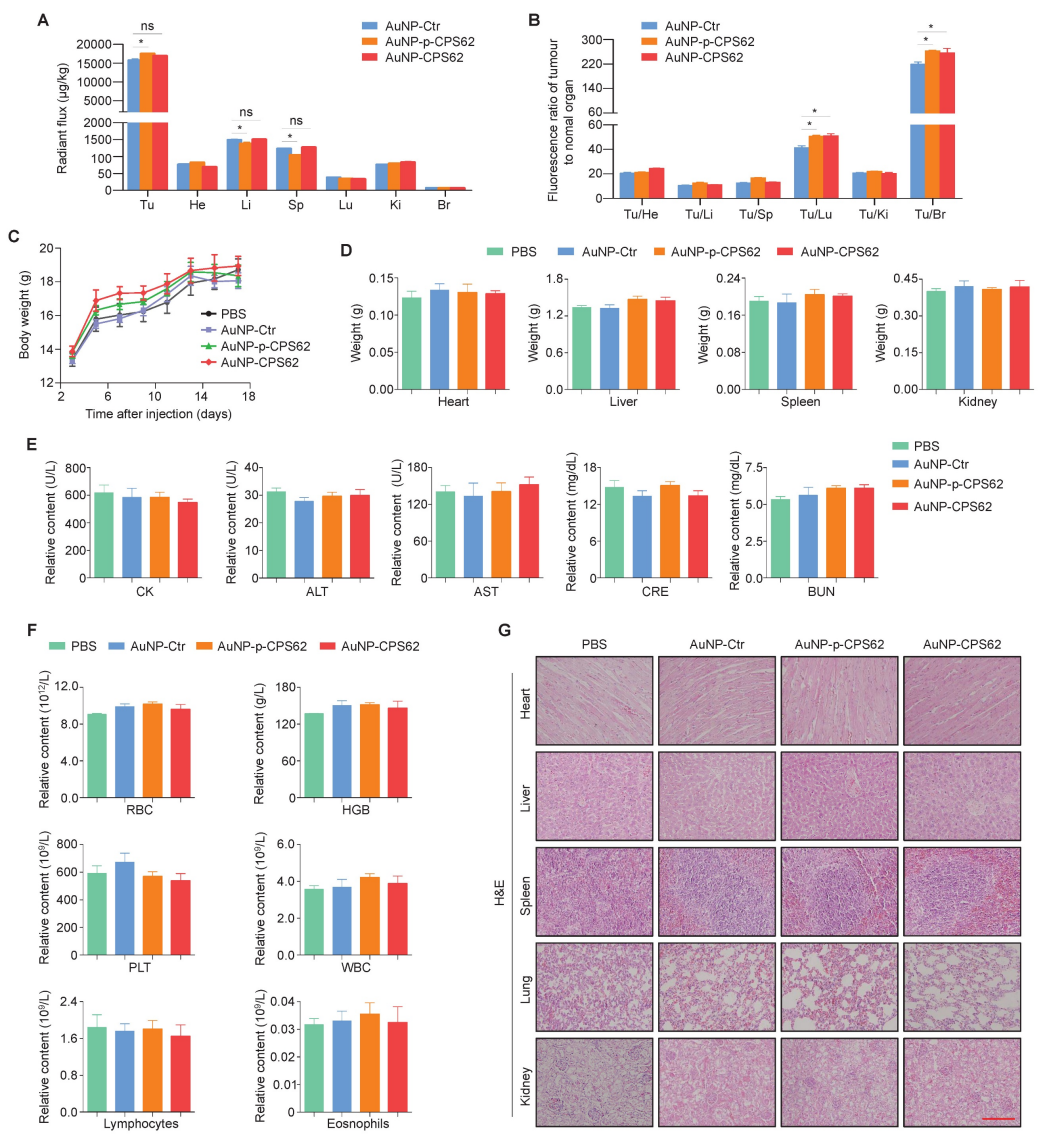
** The impact of polypeptides**-**gold nanoparticles on the biosafety of mice. (A)** Ex vivo semi-quantitative analysis of polypeptides-gold nanoparticles biodistribution after the course of intraperitoneal injection. AuNP enrichment of each organ in mice bearing 8305C xenograft tumors were shown with radiant flux, Tu: tumor; He: heart; Li: liver; Sp: spleen; Lu: lung; Ki: kidneys; Br: Brain. Data were shown as means ± SD. *, *P* <0.05 (n = 3). **(B)** Ex vivo biodistribution analysis of polypeptides-gold nanoparticles treatment. All the multiple of the tumor/organ enrichment were presented as means ± SD. *, *P* <0.05 (n = 3). **(C)** Changes in body weight of mice with xenograft tumors upon various treatments. PBS was used as a blank control. Shown the data as means ± SD (n = 3).** (D)** Mice heart, liver, spleen and kidney weight after 17 days treatment. PBS was used as a blank control. Data were shown as means ± SD (n = 3). **(E)** Measurement of heart indicators (CK), two liver enzymes (ALT and AST) and renal indicators (CRE and BUN) in mice blood after the 17 days treatment. PBS was used as a blank control. Data were presented as mean ± SD (n = 3). **(F)** The count of RBC, hemoglobin, platelet, WBC, lymphocytes and eosinophils in mice blood. PBS was used as a blank control. Shown the data as mean ± SD (n = 3). **(G)** Representative H&E staining of heart, liver, spleen, lung and kidney sections from mice after 17 days treatment. scale bar, 100 µm. Statistical significance between groups was assessed using a two-sided unpaired Student's t-test, except for panel (C), where one-way ANOVA was applied.

We next evaluated the safety of peptide-gold nanoparticles *in vivo*. Firstly, their bio-distributions in tumor sites and normal organs were examined after 17 days of intraperitoneal injection. The semi-quantitative results showed that the AuNPs were mainly enriched in heart, liver, spleen, lung, kidney, brain, especially in tumors of mice (Figure 9A). However, compared with other organs, AuNPs were enriched 10 to 280-fold in tumor sites across all three treatment groups (Figure 9B), indicating that the majority of peptide-gold nanoparticles were accumulated at the tumor sites. We also injected Cy5.5-labeled AuNP-Ctr, AuNP-p-CPS62 or AuNP-CPS62 into mice, and found that the fluorescence signal of nanoparticles was most significantly enriched in the tumor sites compared to other organs (Figure S11), further supporting our conclusion. In addition, there were no significant differences in body weights (Figure 9C) and organ weights (Figure 9D) among PBS-, AuNP-Ctr-, AuNP-p-CPS62- and AuNP-CPS62-treated mice. Next, we assessed the effects of the above treatments on the function of heart, liver and kidney by detecting serum levels of creatine kinase (CK), alanine aminotransferase (ALT), aspartate aminotransferase (AST), creatinine (CRE) and urea (BUN), and failed to find significant differences among different groups (Figure 9E). Moreover, we also analyzed the counts of RBCs, hemoglobin, platelet, WBCs, lymphocytes and eosinophils in the blood. The results showed that no significant hematologic toxicities were observed in any treated mice (Figure 9F). Finally, we evaluated histological changes of organs by H&E staining. As expected, AuNP-Ctr, AuNP-p-CPS62 or AuNP-CPS62 had little adverse effects on the histology of heart, liver, spleen, lung and kidney (Figure 9G). These data suggest that peptide-gold nanoparticles have good biosafety.

## Discussion

The patients with *BRAF^V600E^/pTERT* double mutated tumors, such as thyroid cancer and melanoma, have poor prognosis and high recurrence rate 18-20. Furthermore, these patients showed poor response to conventional radiotherapy, chemotherapy and targeted therapies 19. For example, the emerging dual blocking therapies of BRAF/MEK kinase inhibitors such as Darafenib in combination with Trametinib 47, 48, and triple blocking therapies of BRAF/MEK kinase inhibitors with EGFR monoclonal antibody such as Darafenib in combination with Trametinib and Cetuximab 49 have been widely used in the treatment of the patients with *BRAF^V600E^/pTERT* double mutated tumors. However, their clinical benefits are limited. These are attributed to the vague molecular regulatory mechanism between *BRAF^V600E^* and *pTERT* mutations. Thus, it is necessary to thoroughly clarify the regulatory mechanism between them, and to develop new therapeutic strategies for *BRAF^V600E^/pTERT* double mutated tumors. As reported, BRAF^V600E^ can positively regulate TERT and c-Myc by activating ERK signaling 21, 31. This was also validated by our data showing that BRAF kinase inhibitor PLX4032 significantly down-regulated protein and mRNA levels of TERT and c-Myc in *BRAF^V600E^/pTERT* double mutated cancer cells 8305C and A375. Moreover, we found that TERT knockdown substantially reduced mRNA and protein levels of BRAF, but only down-regulated the protein levels of c-Myc with little effect on its mRNA levels. These findings suggest reciprocal regulation between BRAF and TERT. On the other hand, the regulatory effect of TERT on c-Myc is at the post-transcriptional level. TERT has been evidenced to stabilize c-Myc proteins by binding to its MBI domain 32. However, the MBI domain of c-Myc contains two sites Ser-62 and Thr-58, which can be phosphorylated by ERK and GSK3β, respectively, thereby affecting its protein stability 28. In the present study, we demonstrated that TERT only bonds to pS62-c-Myc but not pT58-c-Myc in 8305C and A375 cells by Co-IP assays, thereby protecting c-Myc from degradation by inhibiting the ubiquitin-proteasome pathway. In turn, knocking down c-Myc in 8305C and A375 cells also down-regulated the expression of BRAF and TERT. To be consistent with this, TERT can be transcriptionally activated by c-Myc 22. In addition, we also evidenced that *BRAF* was a downstream target of c-Myc by dual-luciferase reporter and ChIP assays. These data imply that BRAF, TERT and c-Myc can regulate each other to strengthen their oncogenic functions in *BRAF^V600E^/pTERT* double mutated tumors, and c-Myc plays a central role in this process.

In addition to the fact that TERT interacts with and stabilizes c-Myc, activated ERK and AKT cascades can also prevent the ubiquitination and degradation of p-β-catenin and c-Myc by phosphorylating GSK3β, facilitating β-catenin-mediated *c-Myc* transcription 44. Thus, we next aimed to determine whether c-Myc or TERT is involved in regulating the crucial pathways related to c-Myc degradation. Our data confirmed that c-Myc and TERT knockdown evidently increased the expression of PP2Ac, while reduced the levels of pS62-c-Myc, p-GSK3β, β-catenin and p-AKT^T308^. PP2A is a potent protein phosphatase that contains the C catalytic subunit PP2Ac, which dephosphorylate pS62-c-Myc, p-GSK3β, p-ERK and p-AKT^T308^ in cancer cells 45. Considering that c-Myc knockdown strikingly up-regulated the protein and mRNA levels of PP2Ac, we thus assumed that *PP2Ac* may be a downstream target of c-Myc. This was validated by the data from the dual-luciferase reporter and ChIP assays. Conclusively, c-Myc negatively regulates the transcription of *PP2Ac* to inhibit its dephosphorylation function, thereby maintaining c-Myc protein stability.

Tumor targeted therapy is a promising and precise therapy that targets specific protein or gene 50. Considering that c-Myc plays a vital role in the mutual regulation between BRAF^V600E^ and TERT, we thus propose that targeted therapy against c-Myc may be a potential strategy for the treatment of *BRAF^V600E^/pTERT* double mutated tumors. However, unfortunately, c-Myc has been long considered “undruggable” due to the lack of pharmacologically targetable pockets 29. As TERT can enhance its protein stability by interacting with c-Myc, and the peptide drugs are the most important and effective means to block protein-protein interaction (PPI). Thus, in this study, we designed two peptides which consist of 20 amino acids in the c-Myc/TERT interaction domain to block their interaction for inducing c-Myc degradation. These two peptides contain an N-terminal acetylation, a non-phosphorylated threonine at position 58, a C-terminal cysteine and either a phosphorylated or an unphosphorylated serine at position 62 (pS62 and S62). Although peptide drugs have many advantages, they still have some inevitable disadvantages, such as poor *in vivo* stability and weak membrane penetrating ability 38. AuNPs are widely used in drug delivery to avoid drug premature degradation and achieve drug targeted release in the tumor sites 35. Thus, we constructed the peptide-gold nanoparticles (AuNP-p-CPS62 and AuNP-CPS62) to overcome the above-mentioned disadvantages of peptides, aiming to induce c-Myc degradation by competitively binding with TERT.

On the one hand, we proved that AuNP-p-CPS62 and AuNP-CPS62 indeed blocked the interaction between TERT and c-Myc. On the other hand, using cycloheximide chase and protein ubiquitination assays, we demonstrated that AuNP-p-CPS62 and AuNP-CPS62 induced the degradation of c-Myc proteins by increasing their ubiquitylation levels. In addition, we found that AuNP-p-CPS62 and AuNP-CPS62 could also down-regulate the levels of TERT, BRAF, p-ERK, p-GSK3β and β-catenin, whereas increase the levels of PP2Ac, further activating the signaling pathways associated with c-Myc degradation. These results provide evidence that peptide-gold nanoparticles can effectively promote c-Myc degradation and break the complex regulatory network among BRAF^V600E^, c-Myc and TERT in *BRAF^V600E^/pTERT* double mutated cancer cells.

As *pTERT* mutations in malignancies are promising prognostic biomarkers and the therapeutic targeting of TERT faces drug resistance 51, especially in gliomas 52. While, the oncogene c-Myc is extremely activated in glioma and many other cancers 25. Definitely, we demonstrated the regulatory relationship between TERT and c-Myc in cells with *BRAF^V600E^* and *pTERT* double mutation or single mutation. Thus, blocking the interaction between TERT and c-Myc by AuNP-p-CPS62 or AuNP-CPS62 should have therapeutic effects in tumors with *BRAF^V600E^* and *pTERT* single mutation, even in tumors with no mutation.

As supported, a series of *in vitro* and *in vivo* functional experiments demonstrated that AuNP-p-CPS62 and AuNP-CPS62 had potent anti-tumor activity, reflected by inhibition of cell proliferation, colony formation and tumorigenic ability in nude mice, and induction of cell apoptosis, especially the former. Furthermore, we also proved that AuNP-p-CPS62 and AuNP-CPS62 exhibited excellent tumor targeting and biosafety.

In summary, the present study demonstrates that there exists a complex regulatory network among BRAF^V600E^, c-Myc and TERT in *BRAF^V600E^/pTERT* double mutated tumor cells, and we design two peptides to break the above process by inducing c-Myc degradation. Moreover, to fully realize the potential of peptide drugs, we develop the peptide-gold nanoparticles and demonstrate their potent *in vitro* and *in vivo* anti-tumor activities and excellent biosafety. Thus, blocking the interaction between TERT and c-Myc may overcome the treatment bottleneck of *BRAF^V600E^/pTERT* double mutated tumors, and even become an effective therapeutic option for c-Myc overexpression tumors.

## Supplementary Material

Supplementary figures and tables.

## Abbreviations

ALT: Alanine aminotransferase
AST: Aspartate transaminase
ATC: Anaplastic thyroid carcinoma
AuNP: Aurous nanoparticle
BRCA: Breast cancer
BUN: Blood urea nitrogen
CBC: Complete blood count
CHX: Cycloheximide
CK: Creatine kinase
Co-IP: Co-Immunoprecipitation
CPS62: c-Myc peptides of serine 62
CRE: Creatinine
EPR: Enhanced Permeability and Retention Effect
FTC: Follicular thyroid carcinoma
GTEx: Genotype-Tissue Expression dataset
HPLC: High performance liquid chromatography
HGB: Haemoglobin
LSCM: Laser scanning confocal microscopy
p-CPS62: Phosphorylated c-Myc peptides of serine 62
PLT: platelet
PPIs: Protein-protein interactions
pS62-c-Myc: Phosphorylated c-Myc at serine 62
pT58c-Myc: Phosphorylated c-Myc at threonine 58
TERT promoter): pTERT
RBC: Red blood cell count
Ser62: Serine 62
TAD: Transcriptional activation domain
TCGA: The Cancer Genome Atlas dataset
TEM: Transmission electron microscope
TERT: Telomerase reverse transcriptase
THCA: Thyroid cancer
Thr58: Threonine 58
WBC: White blood cell
