# Supplementary material for: Blocking interplay between TERT and c-Myc: a new therapeutic strategy for BRAFV600E/pTERT double mutated tumors
Source: Int J Biol Sci. 2025 Jul 28;21(11):4961–78. doi: 10.7150/ijbs.111224 (PMC12374824; doi:10.7150/ijbs.111224)
Supplement: Supplementary file 1 — Supplementary figures and tables. [file ijbsv21p4961s1.pdf]

Supplementary Information

Table S1. The primer sequences used in this study for qRT-PCR

Genes	RefSeq ID	Forward primer (5′-3′)	Reverse primer (5′-3′)	Length
c-Myc	NM_002467.5	CACCGAGTCGTAGTCG AGGT	TTTCGGGTAGTGGAAA ACCA	93bp
TERT	NM_198253.2	CGTGGTTTCTGTGTGG TGTC	CCTTGTCGCCTGAGGA GTAG	214bp
BRAF	NM_004333.5	ATTTGGGCAACGAGA CCGAT	GTTGATCCTCCATCAC CACGA	117bp
18S	NR_003142.4	CGCCGCTAGAGGTGAA ATTC	CTTTCGCTCTGGTCCG TCTT	52bp
PP2Ac	NM_002715.4	GGTGGTCTCTCGCCAT CTATAG	CTGGATCTGACCACAG CAAGTC	109bp

Table S2. The antibodies used in this study

Antibodies	Catalog#	Source
anti-c-Myc for WB	sc-764	(Santa Cruz)
anti-c-Myc for IHC	sc-40	(Santa Cruz)
anti-TERT	NB-100-317	(Novus)
anti-BRAF	sc-9002	(Santa Cruz)
anti-p-ERK	#4370	(Cell signaling)
anti-t-ERK	#4695	(Cell Signaling)
anti-PP2Ac	sc-80665	(Santa Cruz)
anti-pT58 c-Myc	#11034	(Signalway Antibody)
anti-pS62 c-Myc	#11311	(Signalway Antibody)
anti-p-GSK3β (S9)	#9323P	(Cell Signaling)
anti-GSK3β	#12456P	(Cell Signaling)
anti-β-catenin	ab32572	(Abcam)
anti-p-Akt308	BS4009	(Bioworld Technology)
anti-t-Akt	BS1810	(Bioworld Technology)
anti-IgG	ab6715	(Abcam)
anti-Ubiquitin	ab33893	(Abcam)
anti-GAPDH	M20006	(Abmart)
anti-Ki67	Cat550609	(BD Pharmingen)

Abbreviations: IHC, immunohistochemistry; WB, western blotting

Table S3. siRNA sequences used in this study

siRNAs	Sequence (5'-3')	Source
si-c-Myc (sense)	AACGATTCCTTCTAACAGA	Ribobio
si-c-Myc (antisense)	UCUGUUAGAAGGAAUCGTT	Ribobio
si-TERT (sense)	GGCCGATTGTGAACATGGA	Ribobio
si-TERT (antisense)	UCCAUGUUCACAAUCGGCC	Ribobio
si-BRAF (sense)	CCTCAAGAGTAATAATATATT	Ribobio
si-BRAF (antisense)	AATATATTATTACTCTTGAGG	Ribobio
si-NC (sense)	UUCUCCGAACGUGUCACGUTT	Ribobio
si-NC (antisense)	ACGUGACACGUUCGGAGAATT	Ribobio

Table S4. The primer sequences used in this study for plasmid construction

Plasmid	Forward primer (5′-3′)	Reverse primer (5′-3′)	Restriction sites
pcDNA3.1(-)A-c-Myc	TGCTGGATATCTGCAGAATTCATGCCCCTCAACGTTAGCTTC	CTTGGTACCGAGCTCGGATCCCTTACGCACAAGAGTTCCGTA	*EcoRI* and *BamH* I

Table S5. The primers used in this study for luciferase reporter plasmid construction

Plasmid	Forward primer (5′-3′)	Reverse primer (5′-3′)	Restriction sites
pGL3-BRAF-Luc	cgagctcttacgcgtgctagcCCGGCCTACAATGTTCATCTTAT	acttagatcgcagatctcgagCCACCGCCACCGCTCAGCGCCG	*NHE1* and *Xho* I
pGL3-PP2Ac-Luc	cgagctcttacgcgtgctagcGCCAAGCCAGTCGGCCTT	acttagatcgcagatctcgagGAACACCTTCTCGTCCAT	*NHE1* and *Xho* I

Table S6. The primers used in this study for ChIP assay

Gene	Position	Forward primer (5’-3’)	Reverse primer (5’-3’)	Length
BRAF	P1: -582/-436	AGTAGTATTGGCTTCCCCGC	CCATGGCTCCGGTTTCCTAC	146bp
	P2: -718/-562	AAAGAAAACACGCGTCGCAC	GCGGGGAAGCCAATACTACT	156bp
	P3: -980/-869	CTGTTCATGAAAGGCACAAAGTA	CCTGTACAAGGCACATAGCATTC	111bp
	P4: -1157/-1015	TCATTCCCGTTTCTCTCTTCCTTA	AACAATTGGGAGAGAAGAGGTCA	142bp
	P5: -1499/-1378	CCGGCCTACAATGTTCATCTTAT	CCTGGGAGCTGATGCTTACTC	121bp
PP2Ac	P1: -143/0	GGTGAGAGCCAGCGGGCCA	GATGCCACCCGCCCCAGCC	143bp
	P2: -786/-642	CCAGAGGTGGGGGTGGTTAA	TGGCAGTCCGATCACGGAAA	144bp
	P3: -1161/-941	GCTCTCTCTTTTAAGCACGT	TTCTTTGGGCCTCGGACCGC	220bp
	P4: -1689/-1489	CCAAGCCAGTCGGCCTTGCC	ACCCCACCCTCAGTATCAGG	200bp

Table S7. The peptide sequences used in this study

Peptides	Sequences (5’-3’)
AuNP-p-CPS62	AC-KKFELLPTPPL{pS}PSRRSGLC-NH2
AuNP-CPS62	AC-KKFELLPTPPLSPSRRSGLC-NH2

Table S8. The IC50 values of AuNPs

	**8305C**		**A375**	
	Mean	SD	Mean	SD
AuNP-Ctr	29.39	2.3	32.25	2.0
AuNP-p-CPS62	16.54	1.8	16.58	1.4
AuNP-CPS62	20.69	1.2	24.14	2.2

Supplementary Figures

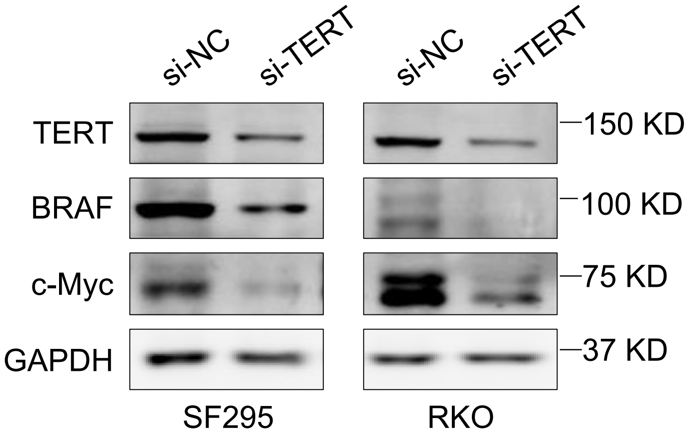

Figure S1. TERT was knocked down in SF295 and RKO cells by siRNAs, and protein expression of TERT, BRAF and c-Myc were measured by western blotting analysis. GAPDH was used as a loading control.

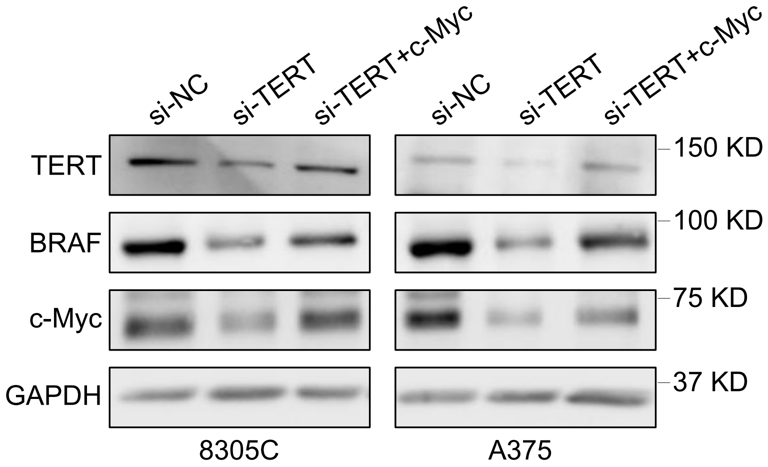

Figure S2. c-Myc was ectopically expressed in TERT-knockdown 8305C and A375 cells, and western blotting analysis was used to evaluate the effect of the above treatments on the expression of TERT, BRAF and c-Myc. GAPDH was used as a loading control.

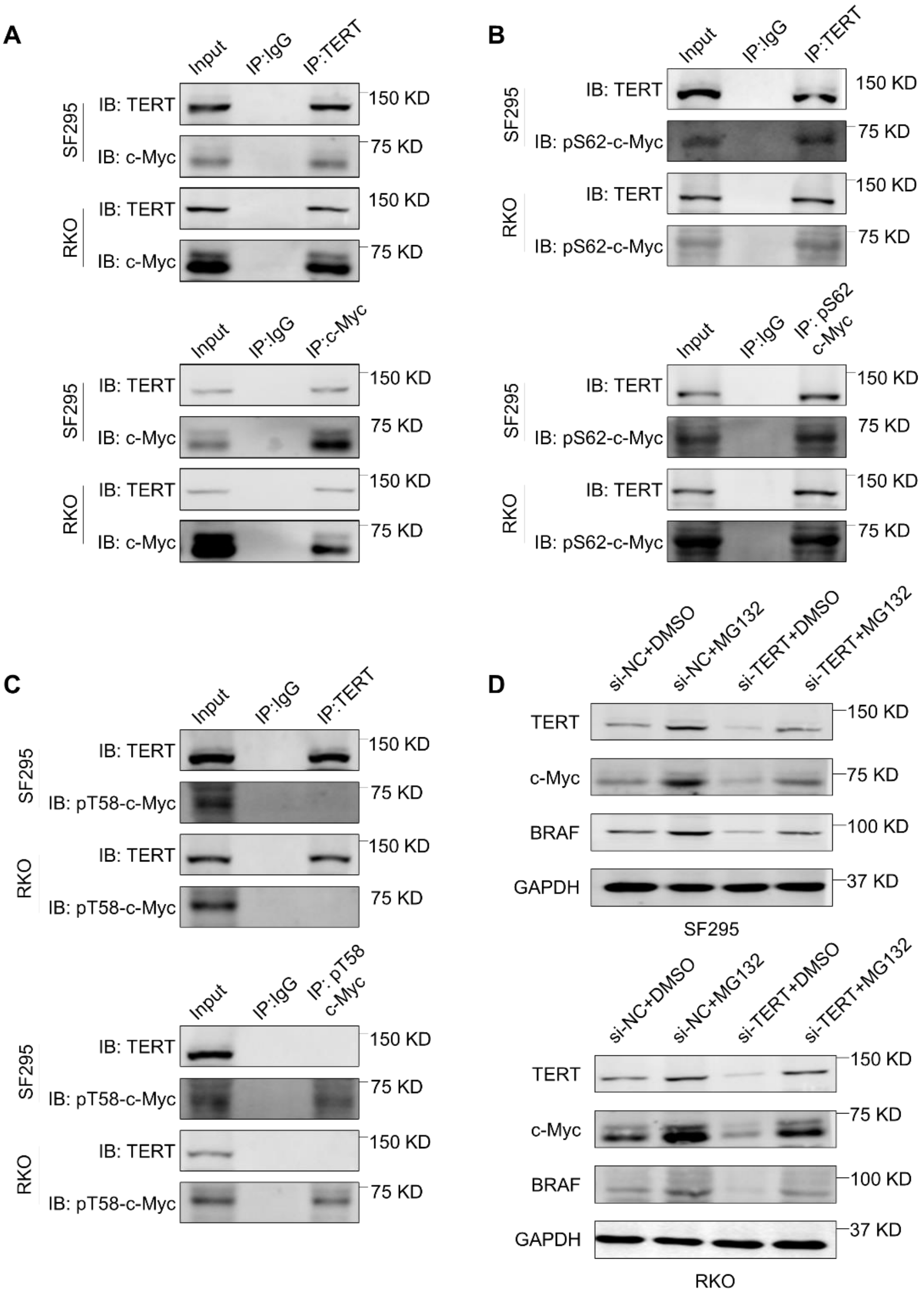

Figure S3. TERT interacts with and stabilizes c-Myc. (A) Reciprocal Co-IP assays were performed in SF295 and RKO cells to determine the interaction between TERT and c-Myc using the indicated antibodies. **(B)** Reciprocal Co-IP assays were performed in SF295 and RKO cells to determine the interaction between TERT and pS62c-Myc using the indicated antibodies. **(C)** Reciprocal Co-IP assays were performed in SF295 and RKO cells to determine the interaction between TERT and pT58c-Myc using the indicated antibodies. The antibody IgG was used as

negative control, and the co-immunoprecipitation is representative of three independently preformed experiments. (**D**) TERT-knockdown SF295 and RKO cells and their control cells were pretreated with 25 μM MG132 or DMSO for 4 h, and western blotting analysis was then used to detect the expression of TERT, BRAF and c-Myc. GAPDH was used as control.

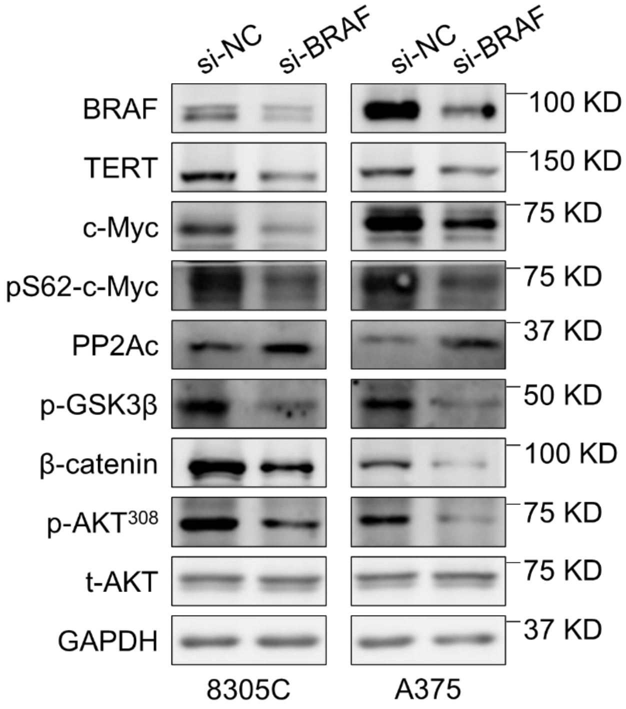

Figure S4. BRAF was knocked down in 8305C and A375 cells, and the expression of TERT, c-Myc, pS62c-Myc, PP2Ac, p-GSK3β, β-catenin and p-AKT^{T308} were determined by western blotting assays. GAPDH was used as a loading control.

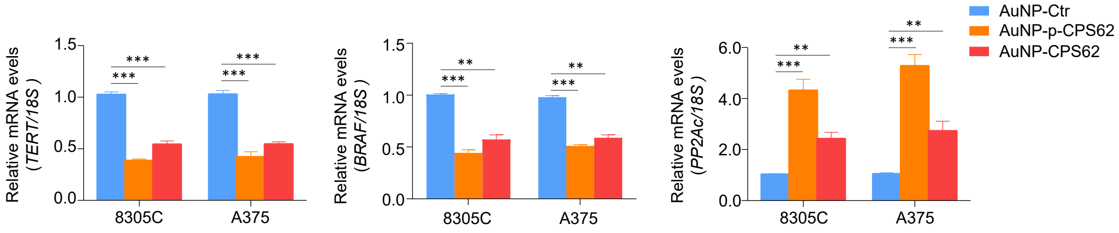

Figure S5. Upon treatment of 8305C and A375 cells with AuNP-Ctr, AuNP-p-CPS62 and AuNP-CPS62, mRNA expression of *TERT*, *BRAF* and *PP2Ac* were determined by qRT-PCR assays. *18S*

rRNA was used as a normalized control. Data were shown as mean ± SD. **, P <0.01; ***, P <0.001 (n = 3).

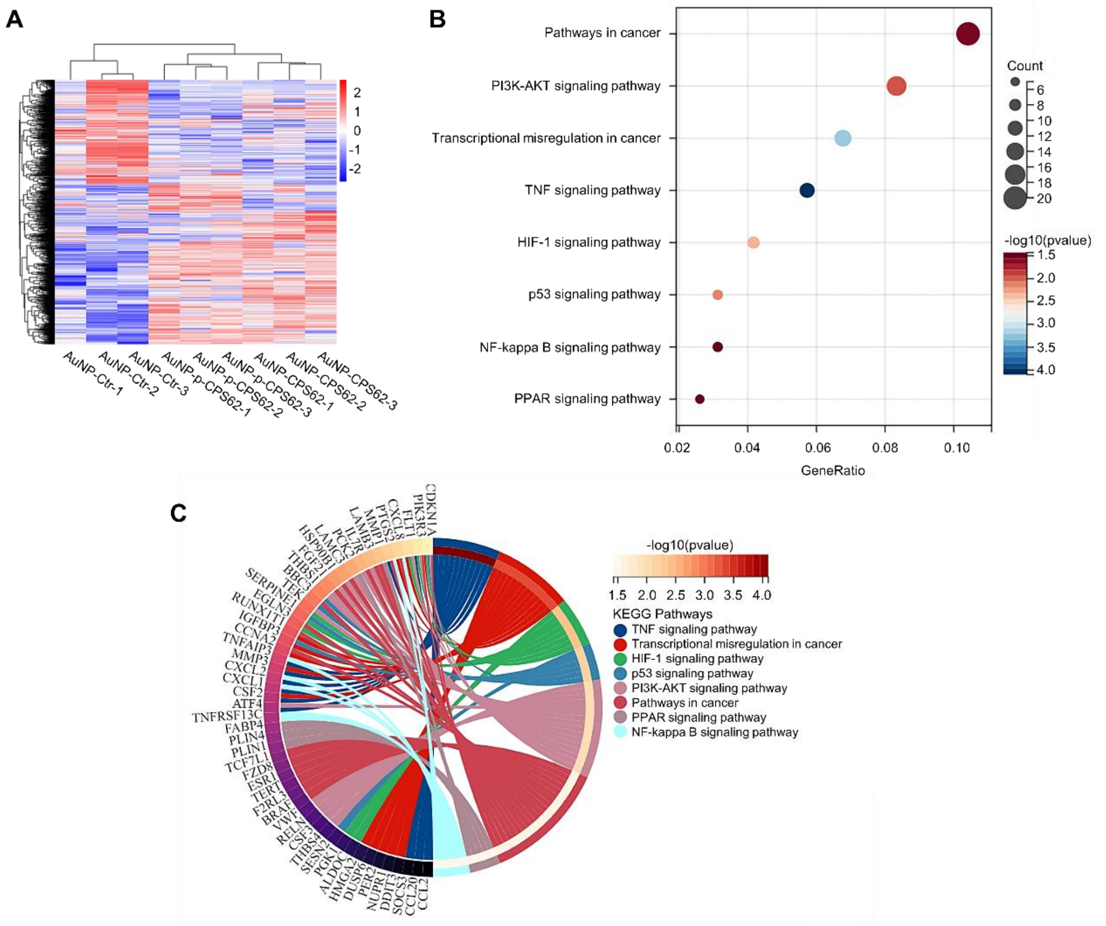

Figure S6. AuNP-p-CPS62 or AuNP-CPS62 treatment causes a significant change in gene expression profiles. (A) Heatmaps showing the overlap of different expression genes in 8305C cells treated with AuNP-Ctr, AuNP-p-CPS62 or AuNP-CPS62. White represents the mean expression of significantly different genes, the redder means the higher the expression, the bluer means the lower the expression, each row represents a gene, and each column represents a sample. **(B)** The 476 DEGs were entered into the SangerBox analysis tool to enrich them onto the corresponding KEGG pathways. Shown were the top eight KEGG pathways enrichment analyses of DEGs (P < 0.05). False discovery rate (FDR) <0.25 were considered statistically significant. **(C)** Shown was the circle diagram of the pathways and the corresponding DEGs (P < 0.05).

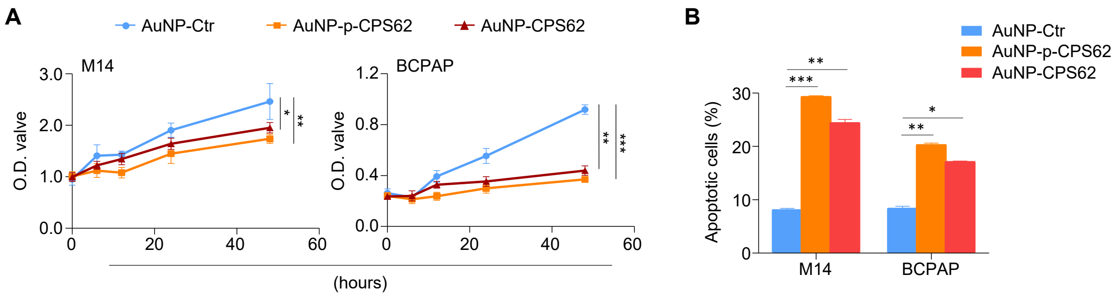

Figure S7. M14 and BCPAP cells were treated with AuNP-Ctr, AuNP-p-CPS62 or AuNP-CPS62, and their effects on cell viability (**A**) and apoptosis (**B**) were determined by MTT assay and flow cytometric analysis, respectively. Data were presented as mean ± SD (cell viability: n =5; cell apoptosis: n =3). *, P <0.05; **, P <0.01; ***, P <0.001.

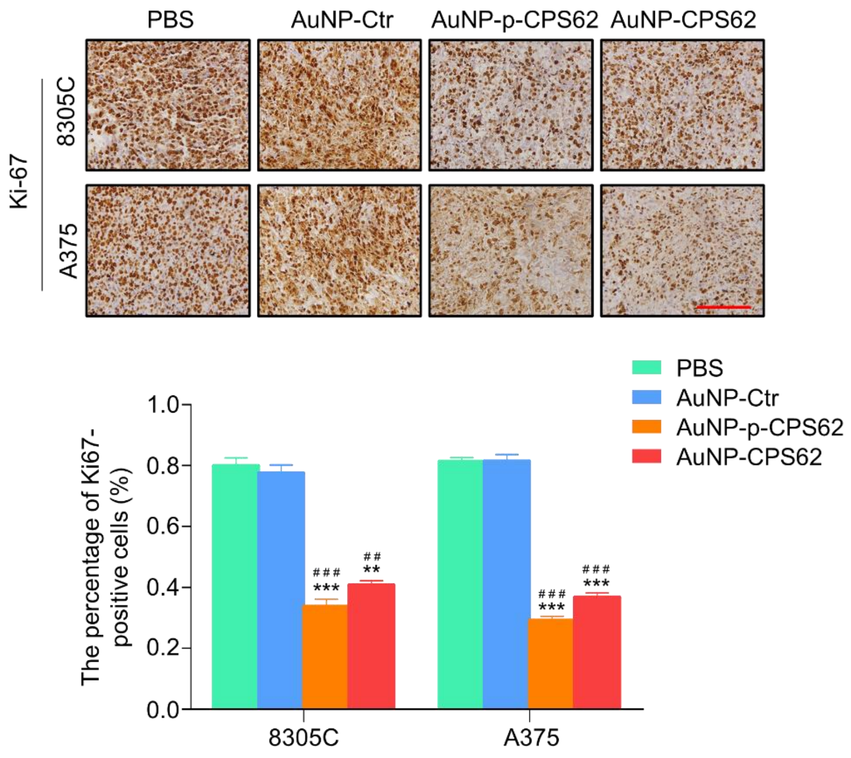

Figure S8. Representative immunohistochemistry staining of Ki-67 in the indicated xenograft tumors (upper panels). Scale bars, 200 μm. The lower panel represents the number of Ki-67-positive cells from five microscopic fields in each group. Data was presented as means ± SD (n =5). # was

used to show the significant difference between PBS and AuNP-p-CPS62 or AuNP-CPS62. * was used to show the significant difference between AuNP-Ctr and AuNP-p-CPS62 or AuNP-CPS62. ##, $P < 0.01$; ###, $P < 0.001$; **, $P < 0.01$; ***, $P < 0.001$.

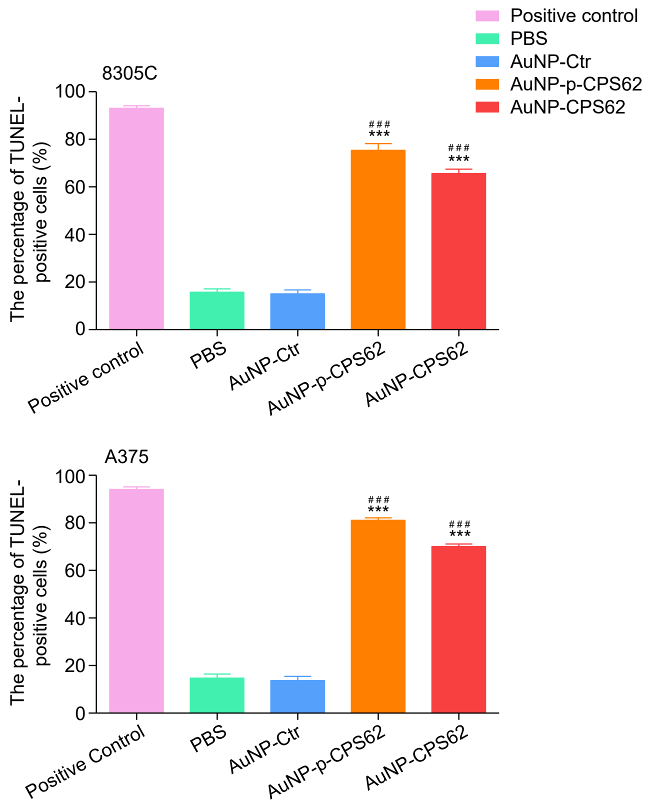

Figure S9. The percentage of TUNEL-positive cells shown in Figure 7E from five microscopic fields in each group. Data were shown as mean ± SD (n =5). # was used to show the significant difference between PBS and AuNP-p-CPS62 or AuNP-CPS62. * was used to show the significant difference between AuNP-Ctr and AuNP-p-CPS62 or AuNP-CPS62. ###, $P < 0.001$; ***, $P < 0.001$.

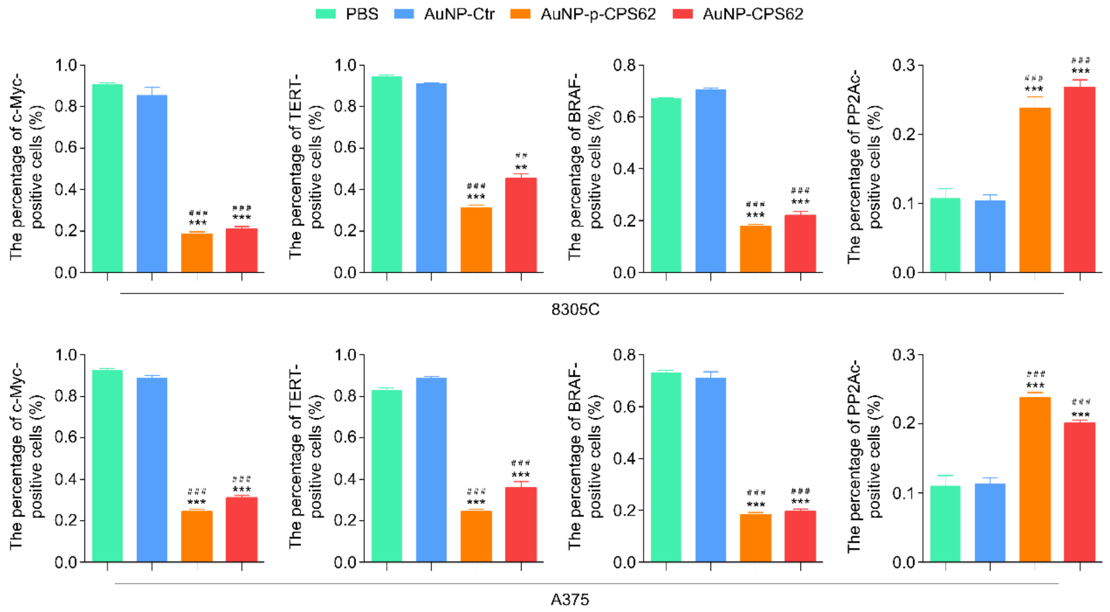

Figure S10. The number of IHC-positive cells, as shown in Figure 7F and Figure 7G, from five microscopic fields in each group. Data were presented as mean ± SD (n =5). # was used to show the significant difference between PBS and AuNP-p-CPS62 or AuNP-CPS62. * was used to show the significant difference between AuNP-Ctr and AuNP-p-CPS62 or AuNP-CPS62. $^{##}$, *P* < 0.01; $^{###}$, *P* < 0.001; **, *P* < 0.01; ***, *P* < 0.001.

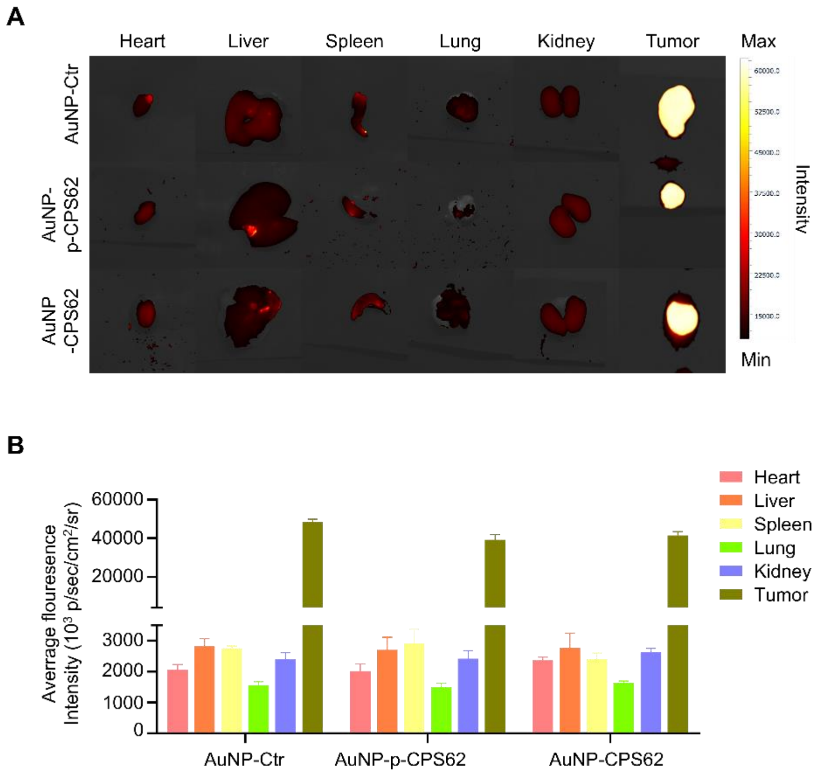

Figure S11. ***In vivo*** **biodistribution of AuNPs.** (**A**) *Ex vivo* fluorescence imaging of major organs and tumor tissues from the indicated mice. (**B**) *Ex vivo* fluorescence radiance of major organs and tumor tissues according to the region of interest (ROI) measurement.
